# Role of the Mitochondrial Protein Import Machinery and Protein Processing in Heart Disease

**DOI:** 10.3389/fcvm.2021.749756

**Published:** 2021-09-28

**Authors:** Fujie Zhao, Ming-Hui Zou

**Affiliations:** Center for Molecular and Translational Medicine, Georgia State University, Atlanta, GA, United States

**Keywords:** mitochondrial protein import machinery, heart disease, TOM complex, TIM23 complex, TIM22 complex, CHCHD4 (MIA40)

## Abstract

Mitochondria are essential organelles for cellular energy production, metabolic homeostasis, calcium homeostasis, cell proliferation, and apoptosis. About 99% of mammalian mitochondrial proteins are encoded by the nuclear genome, synthesized as precursors in the cytosol, and imported into mitochondria by mitochondrial protein import machinery. Mitochondrial protein import systems function not only as independent units for protein translocation, but also are deeply integrated into a functional network of mitochondrial bioenergetics, protein quality control, mitochondrial dynamics and morphology, and interaction with other organelles. Mitochondrial protein import deficiency is linked to various diseases, including cardiovascular disease. In this review, we describe an emerging class of protein or genetic variations of components of the mitochondrial import machinery involved in heart disease. The major protein import pathways, including the presequence pathway (TIM23 pathway), the carrier pathway (TIM22 pathway), and the mitochondrial intermembrane space import and assembly machinery, related translocases, proteinases, and chaperones, are discussed here. This review highlights the importance of mitochondrial import machinery in heart disease, which deserves considerable attention, and further studies are urgently needed. Ultimately, this knowledge may be critical for the development of therapeutic strategies in heart disease.

## Introduction

**Figure 1:**
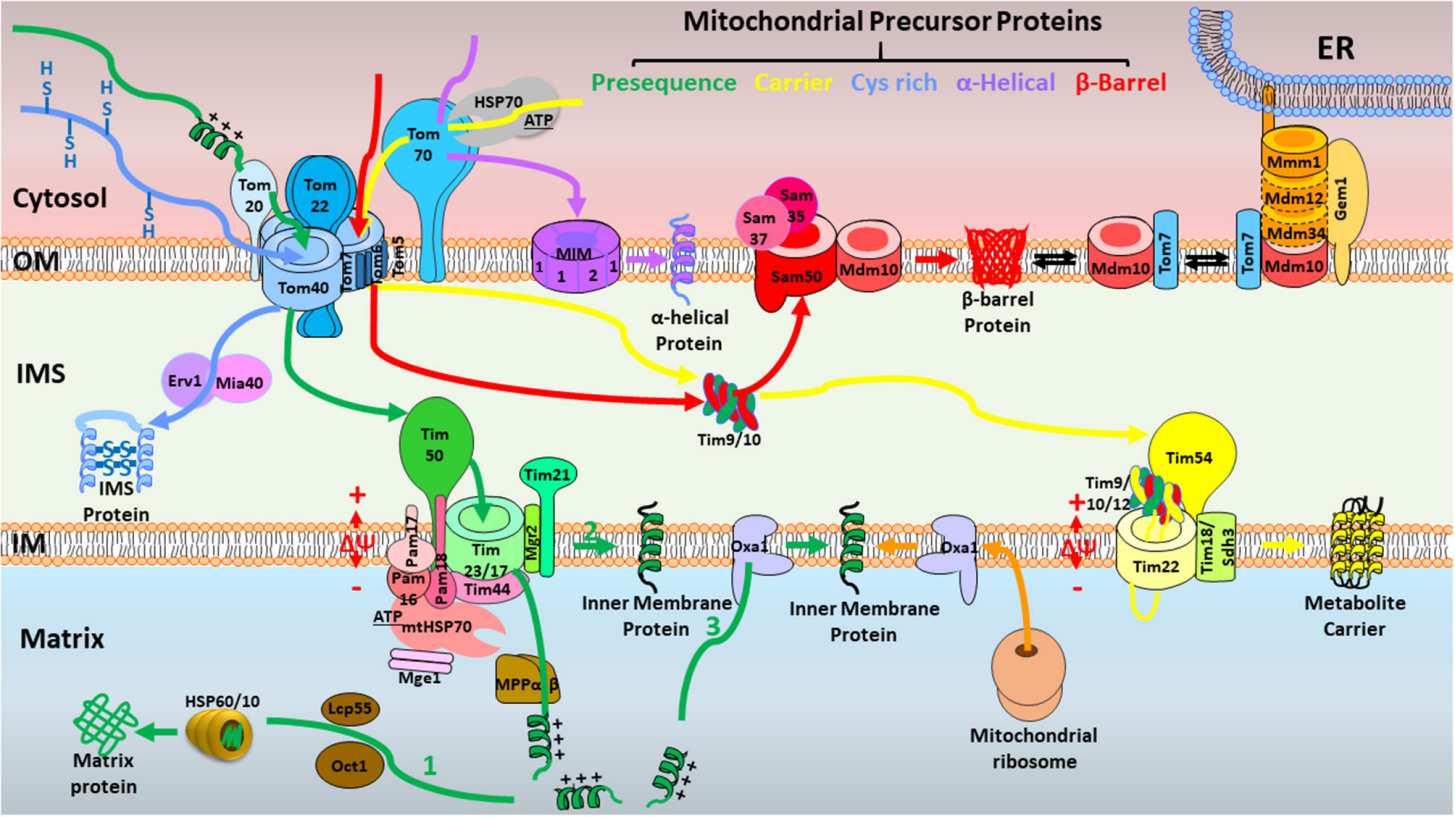
Overview of the major mitochondrial protein import pathways in yeast. First, the presequence pathway transports presequence-carrying cleavable preproteins to the mitochondrial matrix (1) or IM (2, 3) (in green). Presequence-carrying precursors to the mitochondrial matrix (1) are typically recognized by TOM at the OM, passage the IM through TIM23, and are driven into the matrix assisted by PAM. Δψ across the IM is essential for the entry of presequences into the matrix. The presequences are cleaved by MPP, and additional proteolytic processing occurs by intermediate cleaving peptidases. Presequence-carrying precursors that integrate into IM follow two distinct routes. IM proteins are either directly laterally released from the TIM23 complex (2) or transported into the matrix first followed by further insertion into the IM with the help of Oxa1 (3). Oxa1 also is responsible for insertion of IM proteins synthesized on mitochondrial ribosomes. Second, in the carrier pathway for the import of precursor proteins without a cleavable presequence, yet with internal targeting signals into the IM (in yellow), carrier precursors are accompanied by cytosolic chaperones, delivered to the Tom70 receptor of the TOM complex, bound by small TIM chaperones in the IMS, and eventually integrated into the IM by the TIM22 complex in a Δψ-dependent manner. Third, in the MIA pathway for cysteine-rich proteins to the IMS (in blue), the precursors are kept in a reduced state in the cytosol, imported by the TOM complex, oxidized by the MIA machinery, and stay in an oxidized state in the IMS. Fourth, in the SAM complex for β-barrel proteins to the OM (in red), the precursors of β-barrel proteins are imported by the TOM complex at the OM, bound to small TIM chaperones in the IMS, and inserted into the OM by the SAM complex. Fifth, some proteins with α-helical transmembrane segments are inserted into the OM by the MIM complex (in purple). Typically, the Tom40 channel is not involved in this route, but Tom70 is indispensable. In addition, ERMES is a complex that connects ER and mitochondrial OM, facilitating the dynamic substrate exchange between ER and mitochondrion. Mdm10 is a protein with dual localization in SAM and ERMES. OM, outer membrane; IM, inner membrane; IMS, intermembrane space; TOM, the translocase of the outer membrane; TIM23, the inner membrane translocase TIM23; PAM, presequence translocase-associated motor; Δψ, membrane potential; MPP, mitochondrial processing peptidase; Oxa1, oxidase assembly protein 1; TIM22, the carrier translocase of the inner membrane TIM22; MIA, mitochondrial intermembrane space import and assembly machinery; SAM, sorting and assembly machinery; MIM, mitochondrial import complex; ERMES, endoplasmic reticulum (ER)–mitochondria encounter structure.

Mitochondria are vital for energy production in eukaryotic cells, generating cellular ATP through oxidative phosphorylation (OXPHOS) (1). Importantly, mitochondria are also crucial for numerous metabolic pathways, maintenance of calcium homeostasis, and regulation of cell proliferation and apoptosis (2). However, only 13 proteins involved in OXPHOS are encoded by the mitochondrial genome in mammals. About 99%—more than 1,500—mammalian mitochondrial proteins are encoded by the nuclear genome, synthesized as precursors in the cytosol, and need to be imported into mitochondria by mitochondrial protein import machinery (3). To date, six translocases of the mitochondrial protein import machinery have been discovered. The TOM complex serves as the entry gate for most precursors at the outer membrane (OM); the TIM22 and TIM23 complexes at the inner membrane (IM) are responsible for the insertion of carrier precursors into the IM and the translocation of presequence-carrying precursors into the mitochondrial matrix or IM individually; the mitochondrial intermembrane space (IMS) import and assembly machinery (MIA) complex mediates the import of cysteine-rich proteins to the IMS; the SAM and MIM complexes are responsible for insertion of β-barrel proteins and α-helical proteins, respectively, into the OM (Figure 1 and Table 1) (3). The dynamic interaction and cooperation of these mitochondrial protein import pathways enable cells to respond to environmental stress and energy demands rapidly and with plasticity. Further, mitochondrial protein import pathways function not only as independent units for protein translocation, but also are deeply integrated into a functional network of mitochondrial bioenergetics, protein quality control, mitochondrial dynamics and morphology, and interaction with other organelles (4). Mitochondrial protein import deficiency is linked to various diseases, including neuropathies, myopathies, neurodegenerative diseases, cancer, and cardiovascular disorders (5, 6). The heart is a high-energy–requiring organ that depends heavily on mitochondrial activity and the efficient import of mitochondrial proteins. However, in heart disease, the roles of the mitochondrial protein import machinery have not been well-studied. Here, we summarize the current knowledge on mitochondrial protein import in heart disease for the first time. This review highlights the importance of the mitochondrial import machinery in heart disease, which deserves considerable attention, and further studies are urgently needed. Ultimately, this knowledge may be critical for the development of therapeutic strategies in heart disease.

**Table 1:** Components of mitochondrial import machinery in fungi and mammals.

**Complex**	**Fungi (protein/gene name)**	**Mammals (protein/gene name)**
TOM	Tom20/TOM20	Tom20/TOMM20
	Tom22/TOM22	Tom22/TOMM22
	Tom70/TOM70	Tom70/TOMM70
	Tom40/TOM40	Tom40/TOMM40
	Tom5/TOM5	Tom5/TOMM5
	Tom6/TOM6	Tom6/TOMM6
	Tom7/TOM7	Tom7/TOMM7
TIM22	Tim22/TIM22	Tim22/TIMM22
	Tim9/TIM9	Tim9/TIMM9
	Tim10/TIM10	Tim10a/TIMM10A
	Tim12/TIM12	Tim10b/TIMM10B
	Tim54/TIM54	
	Tim18/TIM18	
	Sdh3/SDH3	
		AGK/AGK
		Tim29/TIMM29
TIM23	Tim50/TIM50	Tim50/TIMM50
	Tim23/TIM23	Tim23/TIMM23
	Tim17/TIM17	Tim17a/TIMM17A
		Tim17b/TIMM17B
	Tim21/TIM21	Tim21/TIMM21
PAM	Tim44/TIM44	Tim44/TIMM44
	mtHsp70/SSC1	Mortalin/HSPA9
	(Pam16/Tim16)/ (PAM16/TIM16)	(Tim16/Magmas)/ (PAM16/MAGMAS)
	(Pam18/Tim14)/ (PAM18/TIM14)	(DnaJC15/MCJ)/ DNAJC15
		(DnaJC19/Tim14)/DNAJC19
	(Mge1/GrpE)/GRE1	mtGrpE/GRPEL1
	Pam17/PAM17	
OXA	Oxa1/OXA1	Oxa1/OXA1L
Matrix	(Mas1/β-MPP)/MAS1	β-MPP/PMPCB
	(Mas2/α-MPP)/MAS2	α-MPP/PMPCA
	MIP/OCT1	MIP/MIPEP
	Hsp60/HSP60	Hsp60/HSPD1
	Hsp10/HSP10	Hsp10/HSPE1
	mtHsp70/SSC1	mtHsp70/GRP75
	(Mge1/GrpE)/GRE1	mtGrpE/GRPEL1
MIA	Mia40/MIA40	Mia40/CHCHD4
	Erv1/ERV1	ALR/GFER
		AIF/AIFM1
SAM	Sam50/SAM50	Sam50/SAMM50
	Sam35/SAM35	Metaxin-1/MTX1
	Or	Metaxin-2/MTX2
	Sam37/SAM37	Metaxin-3/MTX3
	Mdm10/MDM10	
Soluble IMS TIMs	Tim9/TIM9	Tim9/TIMM9
	Tim10/TIM10	Tim10a/TIMM10A
	Tim8/TIM8	Tim10b/TIMM10B
	Tim13/TIM13	Tim8a/TIMM8A
		Tim8b/TIMM8B
		Tim13/TIMM13
Cytosolic chaperones	Hsp70/(SSB1 or SSB2)	Hsc70/HSPA8
		Hsp90 alpha/HSP90A
		Tom34/TOMM34
		AIP/AIP

## Overview of Mitochondrial Protein Import Machinery (In Yeast)

### Presequence Pathway to the Mitochondrial Matrix and IM

The presequence pathway is the best characterized pathway, responsible for the import of ~60% of all mitochondrial proteins (7). The precursor proteins in this pathway carry a cleavable N-terminal presequence that functions as a targeting signal (7–9). This unique feature of the pathway distinguishes it from all the others, where the precursor proteins do not have cleavable presequences, but possess different kinds of internal targeting signals. **Presequence-carrying precursors to the mitochondrial matrix** are typically recognized by the translocase of the outer membrane (TOM) (9–11), passaged through the IM by the translocase of the inner membrane (TIM23) (12–16), and driven into the matrix assisted by the presequence translocase-associated motor (PAM) (Figure 1) (17–24). The membrane potential (Δψ) across the IM is essential for the activation of the TIM23 channel and the translocation of presequences into the matrix (25–28). The presequences are cleaved by the mitochondrial processing peptidase (MPP) (3, 29–31), and additional proteolytic processing occurs by intermediate cleaving peptidases (7, 32–34). **Presequence-carrying precursors that integrate into the IM** follow two distinct routes (Figure 1). IM proteins are either directly released laterally from the TIM23 complex (35–37) or transported first into the matrix, followed by further insertion into the IM with the help of the oxidase assembly protein 1 (Oxa1) insertase (38–41). Oxa1 also is responsible for insertion of IM proteins synthesized on mitochondrial ribosomes (42).

**The presequences** are located at the N-termini of preproteins and typically consist of ~15–50 amino acids. An essential characteristic of mitochondrial presequences is the formation of an amphipathic α-helix that is specifically recognized by mitochondrial import receptors and other mitochondrial import components during preprotein translocation by TOM, TIM23, and PAM (3).

**The TOM complex** is the main gate for precursors entering mitochondria. The TOM complex is composed of Tom20, Tom22, and Tom70 as receptors, β-barrel protein Tom40 forming the channel, and three small associated proteins, Tom5, Tom6, and Tom7 (11, 43–45). Tom20 and Tom22 function cooperatively as the receptors for presequence-carrying precursors (9, 10, 46). Tom70 mainly functions as the receptor for preproteins with internal targeting sequences, such as carrier precursors (47–52). Presequence-carrying precursors cross the Tom40 channel as linear polypeptide chains (44, 45, 53–57), and interact with the tail of the Tom22 receptor in the IMS (57). Tom22 has presequence binding sites on both the cytoplasmic and IMS sides of the OM. The exact roles of the three small subunits, Tom5, Tom6, and Tom7, have not been well-clarified. It has been proposed that they are not essential for TOM functions but are involved in the assembly and stability of the TOM complex (58–61).

**The TIM23 complex** translocates cleavable preproteins into mitochondrial matrix or IM. Tim50, Tim23, Tim17, and Tim21 compose the main elements of the TIM23 complex (12, 13, 16, 62–65). Tim50 functions as a presequence receptor that binds preproteins emerging in the IMS (63) and a channel blocker that closes out the TIM23 channel in the absence of preproteins (25, 66–69). Tim23 and associated partner Tim17 form the channel (16, 25, 28, 64, 70, 71). Tim21, Tim50, and Tim23 expose domains to the IMS that transiently connect the TOM and TIM23 complexes to facilitate the preprotein transfer (16, 67, 72–74). Additionally, Tim21 also physically links the TIM23 complex to the respiratory chain III-IV supercomplex [*bc*1 complex and cytochrome *c* oxidase (COX)] (65, 75, 76). Tim21 thus plays a dual role in TOM-TIM23 transfer and the recruitment of respiratory chain complexes. A small membrane protein, Mgr2, functions as a lateral gatekeeper for preproteins that are sorted into the IM (35, 76). The Δψ across the IM is crucial for translocation of the presequences through the Tim23 channel, which is negative at the matrix side and positive at the IMS side of the IM, whereas presequences are mostly positively charged. Two roles have been assigned to Δψ: activation of TIM23 channel (25) and an electrophoretic effect that drives the import of presequences (77, 78).

**PAM**. The Δψ is a prerequisite for translocation of the presequence across the TIM23 channel. Nevertheless, it is not sufficient to import the entire protein into the matrix. PAM is necessary for the translocation of matrix proteins. The core of PAM is formed by the molecular chaperone mitochondrial 70 kDa heat shock protein (mtHsp70) (17, 18) and its co-chaperones (Tim44, Mge1, Pam16, Pam17, and Pam18) (16, 21, 79, 80). mtHsp70 binds the unfolded polypeptide chain and drives its translocation into the matrix in an ATP-dependent manner (17, 18). The peripheral membrane protein Tim44 is a docking site for mtHsp70 at the TIM23 complex (21). Mge1 (also known as mitochondrial GrpE) stimulates the release of ADP from mtHsp70 (81). Pam16, Pam17, and Pam18 are three membrane-associated co-chaperones. Pam18 (also termed Tim14) is a J-type co-chaperone that stimulates the ATPase activity of mtHsp70 (82, 83). The J-related Pam16 (Tim16) forms a complex with Pam18 and functions as a negative regulator (82–86). Pam17 mediates the organization of the TIM23–PAM interaction (79, 87).

**MPP**. Once arriving in the matrix, the presequences of both IM-sorted and matrix-targeted precursors are removed by a heterodimeric enzyme, MPP (3, 29–31, 88). Additional proteases, the intermediate cleaving peptidase (Icp55) (7, 89) and the octapeptidyl aminopeptidase (Oct1, also termed mitochondrial intermediate peptidase, MIP) (90, 91), can remove destabilizing N-terminal amino acid residues of the imported proteins. mtHSP70 and other chaperones, like the HSP60–HSP10 chaperonin complex, further assist proteins folding into their active forms (92). The clipped presequence peptides undergo subsequent degradation by the matrix peptidasome, termed presequence protease (PreP) or Cym1 (93, 94).

**OXA** translocase is vital for exporting proteins from the mitochondrial matrix into the IM. OXA has three different roles. (1) Proteins encoded by the mitochondrial genome are exported into the IM by Oxa1 (42, 95–97). (2) Some presequence-carrying proteins imported into the matrix *via* the TOM-TIM23 machinery are exported into the IM *via* Oxa1. This import-export pathway is termed conservative sorting of nuclear-encoded IM proteins (38–41, 98–100). (3) Oxa1 is also vital for the assembly of the carrier translocase TIM22 (38, 101).

### Carrier Pathway Into the IM

The carrier pathway is the second mitochondrial protein import pathway to be discovered, and is responsible for importing precursor proteins without a cleavable presequence, yet with different kinds of internal targeting signals (3, 102–104). The carrier precursors are accompanied by cytosolic chaperones, such as the HSP70 and HSP90 classes in the cytosol, directly delivered to the Tom70 receptor of the TOM complex (47, 105, 106), and then bound by small TIM chaperones in the IMS (107–110) and eventually integrated into the IM by the carrier translocase of the IM (TIM22) complex in a Δψ-dependent manner (Figure 1) (109, 111–116).

**Chaperone-guided transport of carrier precursors (including chaperones in the cytosol and IMS)**. The carrier import pathway uses the same mitochondrial entry gate as the presequence pathway, the TOM complex. However, the mechanisms of translocation differ significantly. The involvement of cytosolic (47, 105, 106) and mitochondrial IMS chaperones, which is crucial to prevent aggregation of the hydrophobic carrier precursors in the aqueous environment, is the main feature distinguishing this from the presequence pathway. Chaperones of the Hsp70 and Hsp90 classes directly participate in delivering the precursors to Tom70 (47, 105, 106). The receptor Tom70 possesses two distinct binding sites, one for the precursor and another for a chaperone (47, 49, 117), ATP is needed to release the precursor proteins from the cytosolic chaperones (47, 48). Upon binding to Tom70, the carrier precursors are transferred to the central receptor Tom22, followed by insertion into the Tom40 channel in a loop conformation (118, 119), and transferred to small TIM chaperones in the IMS (107–110). These small TIM heterohexameric chaperone complexes, like the Tim9-Tim10 complex (120, 121) and the homologous Tim8-Tim13 complex (122), bind to the precursor proteins and transfer them through the aqueous IMS to IM.

**Insertion of carrier precursors into the IM**. The TIM22 complex consists of the receptor-like protein Tim54, the channel-forming protein Tim22, the Tim9-Tim10-Tim12 chaperone complex, and the Tim18-Sdh3 module. The majority of Tim54 domain is exposed to the IMS and probably functions as the binding site for the Tim9-Tim10-Tim12 complex (123, 124). The Tim9-Tim10-Tim12 complex is a modified form of the IMS chaperone, docking onto the TIM22 complex (123, 125). Carrier precursors are inserted into the Tim22 channel in a Δψ-dependent manner (115). The Tim18-Sdh3 module is involved in the assembly of the TIM22 complex (126, 127). The carrier precursors are first bound to the Tim9–Tim10–Tim12 chaperone complex on the surface of the translocase. Upon activation of the Tim22 channel (Δψ-dependent), the precursors are inserted into the translocase, probably in a loop structure. Finally, the proteins are laterally released into the lipid phase of the IM.

### MIA Complex

Many IMS proteins contain internal targeting signals and characteristic cysteine motifs. In the cytosol, the precursors are kept in a reduced and unfolded state (128). Upon import by the TOM complex (60), they are oxidized by the MIA machinery, and stay in the IMS in an oxidized state (Figure 1). The MIA system consists of two main components: the oxidoreductase Mia40 and the sulfhydryl oxidase Erv1 (129–133).

**Mia40 serves as a receptor and protein disulfide carrier**. Most IMS proteins are synthesized without cleavable presequences but contain cysteine motifs. Unlike presequence-carrying precursors and carrier precursors, none of the Tom receptors is necessary for the import of MIA substrates (60, 61). Instead, upon passage through the Tom40 channel (60), Mia40 functions as a receptor on the IMS side of the Tom40 channel (133–138). It recognizes an internal signal of the precursor proteins, typically consisting of a hydrophobic element flanked by a cysteine residue (133, 139, 140). Mia40 binds to precursors *via* hydrophobic interaction and catalyzes the formation of disulfide bonds in imported proteins (133, 138). The disulfide bonds facilitate the conformational stabilization and assembly of many IMS proteins.

**Erv1 cooperates with Mia40 in a disulfide relay**. Mia40 does not form disulfide bonds *de novo*. Disulfide bonds are generated by Erv1 and transferred to Mia40 by the formation of transient intermolecular disulfide bonds (141). Mia40 then transfers the disulfides onto the imported protein. Upon transfer of disulfide bonds to proteins, cysteines of Mia40 become reduced and are re-oxidized by Erv1. Electrons originating from the oxidation of imported proteins flow in the opposite direction. They flow from Mia40 to Erv1 and then to O_2_ or cytochrome *c* of the respiratory chain (141–144). In addition to most IMS proteins, some IM and matrix proteins are also MIA-system–dependent (28, 71, 113, 114, 145).

### Sorting Pathways of Mitochondrial OM Proteins

All the mitochondrial OM proteins are imported from the cytosol. The membrane contains two different types of integral protein: β-barrel proteins, which are integrated into the membrane by multiple transmembrane β-strands, and α-helical proteins, which are membrane-anchored by one or more α-helical transmembrane segments.

**Sorting and Assembly Machinery for** **β-Barrel Proteins**. The precursors of β-barrel proteins initially pass through the TOM complex at the OM (146), then bind to small TIM chaperones in the IMS (110), like the carrier precursors, to avoid aggregation (Figure 1). Subsequent insertion of the β-barrel proteins into the OM is performed by the SAM complex, which consists of a membrane-integrated protein, Sam50 (Tob55), and two peripheral membrane proteins exposed to the cytosol, Sam35 and Sam37 (147–149). Folding of the β-barrel proteins occurs at Sam50-Sam35, followed by lateral release into the lipid phase of the OM (148, 150, 151). Sam37 directly interacts with Tom22, coupling the TOM and SAM complexes into a transient supercomplex that promotes the efficient transfer of precursor proteins (54, 152).

**OM Insertion of** **α-Helical Proteins**. The main α-helical proteins are classified as signal-anchored proteins (containing an α-helical transmembrane segment at the N-terminus), tail-anchored proteins (containing an α-helical transmembrane segment at the C-terminus), and polytopic (multi-spanning) OM proteins. α-helical OM proteins are imported *via* distinct routes that do not involve the Tom40 channel, in contrast to most mitochondrial proteins. The insertion of some signal-anchored and polytopic OM proteins is performed by the MIM complex (153–156), which consists of multiple copies of Mim1 and one copy of Mim2, both of which are small single-spanning OM proteins (Figure 1) (157, 158). Tom70 is required for the insertion of some polytopic proteins into MIM (155, 156). In the case of tail-anchored proteins and some multi-spanning proteins, import is aided by the lipid composition of the membrane, and no proteinaceous machinery has been identified (159–162). However, the exact mechanism for sorting and insertion of α-helical outer membrane proteins is only partially understood, and further studies are urgently needed.

## Integration of Mitochondrial Protein Import into Functional Networks

Mitochondrial protein import pathways not only function as independent units for protein translocation, but also are deeply integrated into a functional network of mitochondrial bioenergetics, protein quality control, mitochondrial dynamics and morphology, and interaction with other organelles.

**The protein import activity serves as a sensor for the fitness and quality of mitochondria**. The protein import activity is determined by the energetic state (Δψ, ATP levels) and protein homeostasis of mitochondria. Both the translocation of precursor proteins through the TIM23 complex and the TIM22 complex require the Δψ (25, 77, 78, 113, 115, 116). The ATP-dependent chaperones play essential roles in delivering carrier precursors to Tom70 receptor (47, 48), driving presequence precursor translocation to the matrix (17, 18) and folding in the matrix (92). Impairment of respiratory chain activity, reduction of ATP levels, and accumulation of misfolded proteins or reactive oxygen species (ROS) in the matrix (163) will directly affect the import-driving activity of the translocases. The protein import activity of mitochondria is thus a sensitive indicator of their energetic state and fitness.

**Mitochondrial protein import machinery and respiratory chain assembly**. Both the insertion of mitochondrial-encoded proteins from the matrix into the IM and the import of nuclear-encoded precursors from the cytosol into mitochondria rely heavily on the mitochondrial protein import machinery. Increased mitochondrial ROS levels generated by the respiratory chain contribute to decreased mitochondrial translation efficiency (164). In addition, the TIM23 complex forms supercomplexes with respiratory complexes III and IV as well as with the ADP/ATP carrier. These interactions of the TIM23 complex facilitate protein import under energy-limiting conditions (65, 75, 165) and can also promote the assembly of respiratory complexes (166–168). The respiratory chain complexes also function as assembly platforms for some PAM subunits (75).

**Mitochondrial protein import machinery associated with protein quality control, specifically in the following aspects**: (1) Mitochondrial unfolded protein response (UPR^mt^): the stress-activated transcription factor ATFS-1 contains a mitochondrial presequence and a nuclear localization signal. In healthy mitochondria, ATFS-1 is imported into the mitochondrial matrix and degraded by the LON AAA+ protease. When mitochondrial import is mildly impaired, ATFS-1 is translocated into the nucleus, where it functions as a transcription factor and induces expression of mitochondrial chaperones, proteases, and other elements to promote recovery of impaired mitochondria (169–171). (2) Unfolded protein response activated by mistargeted mitochondrial proteins (UPR [am]): upon mild damage to mitochondrial protein import, some mitochondrial precursors fail to enter mitochondria and accumulate in the cytosol, thus triggering mitochondrial Precursor Over-accumulation Stress (mPOS). This is followed by a stress response termed UPR [am] that reduces cytosolic protein synthesis and increases proteasome activity to clear the mistargeted proteins from the cytosol (172, 173). (3) PTEN-induced kinase 1 (PINK1)/Parkin-induced mitophagy: in healthy conditions, PINK1 is imported into the IM by the presequence pathway and processed by MPP and the presenilin-associated rhomboid-like protease PARL, following the retrotranslocation into the cytosol and degradation by the proteasome (174, 175). Upon severe damage to mitochondrial protein import, PINK1 remains at the OM bound to the TOM complex, where it phosphorylates ubiquitin and the E3 ubiquitin ligase Parkin, triggering the removal of damaged mitochondria by mitophagy (176, 177). (4) Mitochondria as guardian in the cytosol (MAGIC): in certain conditions, some aggregation-prone or misfolded cytosolic proteins may be imported into mitochondria for further degradation. This process is termed MAGIC, suggesting a crucial role of mitochondria in cytosolic proteostasis (178). (5) Proteolysis of mitochondrial proteins: upon removal of the presequence by MPP, destabilizing N-terminal amino acid residues of the imported proteins can be further removed by the intermediate cleaving peptidase Icp55 (which removes a single amino acid) or the mitochondrial intermediate peptidase Oct1 (which removes an octapeptide) (7, 34). The matrix AAA+ proteases, CLPXP and LON, degrade misfolded proteins and prevent protein aggregation in the matrix (179–182). The IM contains two AAA+ proteases: the i-AAA protease removes misfolded proteins from the IM, IMS, and OM (183–185), whereas the m-AAA protease degrades proteins from the matrix and IM (186, 187). Thus, the process of mitochondrial protein import is connected to protein turnover and quality control.

**Mitochondrial protein import machinery connected to mitochondrial membrane architecture and dynamics**. The mitochondrial contact site and cristae organizing system (MICOS) is a large protein complex enriched at crista junctions of the IM (188–190). It is crucial for the maintenance of inner membrane cristae organization and is embedded into an interactional network with protein translocases, including TOM, SAM, and MIA. Thus, it provides a dynamic link between protein import, mitochondrial membrane dynamics, and membrane contact sites (136, 188, 189, 191).

The inner-membrane fusion protein optic atrophy (OPA1) is an example of how protein import and processing are connected to mitochondrial membrane dynamics. OPA1 is first processed by matrix MPP, generating a long isoform, and further processed by different IM proteases, AAA+ protease, or OMA1 in mammals and Pcp1 in yeast, yielding a short isoform (192–195). The balance between long OPA1 and the short isoform is essential for membrane fusion and fission, which is modulated by stress and mitochondrial energetic state. Thus, the processing of imported mitochondrial protein is linked to mitochondrial fragmentation, mitophagy, or even cell death.

**Endoplasmic reticulum–mitochondria encounter structure (ERMES)**. ERMES is a multi-subunit protein complex that connects the endoplasmic reticulum and mitochondrial OM, mainly formed by the MDM complex (196, 197). Other molecules, such as voltage-dependent anion-selective channel (VDAC) (198), TOM70 (199, 200), and inositol trisphosphate (inositol 1,4,5-trisphosphate) receptors (194) also play crucial roles in forming ER-mitochondria contact sites. The outer membrane protein Mdm10 is a subunit of both SAM and MDM complexes (196). The shuttling of Mdm10 between SAM and MDM is regulated by the small protein Tom7 (58, 196, 197, 201). Therefore, TOM, SAM, and ERMES are linked as a functional network, involved in the maintenance of mitochondrial morphology and the transport of lipids and calcium (202–205).

## Mitochondrial Protein Import Machinery and Heart Disease

Mitochondrial dynamics have become a key topic in the field of heart disease. However, only limited studies investigated the involvement of mitochondrial protein import machinery in these diseases (Summarized in Table 2). Moreover, most of these studies were related to the presequence pathway, which undertakes the import of ~60% of all mitochondrial proteins.

**Table 2:** Reported proteins or genes in mitochondrial protein import machinery associated with heart disease.

**Protein/gene name**	**Import pathway/role**	**Associated disease/stress or physiological process**	**Expression or function in disease**	**References**
Tom20/ TOMM20	TOM complex/ Receptor	Myocardial I/R injury; Cardiac calcium overload	Decreased level of Tom20 protein upon myocardial I/R injury; Potential regulator of cardiac calcium homeostasis.	(206–210)
Tom22/ TOMM22	TOM complex/ Receptor	Cardiac calcium homeostasis; Cardiac aldosterone synthesis; Chronic hypoxia	Receptor for mitoBK_Ca_; Promoting the synthesis of cardiac aldosterone; Increased level of Tom22 mRNA in chronically hypoxic rat hearts.	(211–215)
Tom70/ TOMM70	TOM complex/ Receptor	Cardiac hypertrophy; Myocardial I/R injury; Myocardial infarction; Diabetic cardiomyopathy; Heart failure	Decreased level of Tom70 protein in hypertrophic and diabetic hearts, upon I/R injury or post-MI; Altered phosphorylation level of Tom70 in rat hearts with heart failure.	(216–228)
Tom40/ TOMM40	TOM complex/ Channel	Cardiovascular-related traits; Cardiac arrhythmia; Heat stress-induced apoptosis; Cardiac aging	TOMM40/APOE locus was associated with the main risk factors for cardiovascular disease; Homozygous deletion of TOMM40 in mammals was lethal; Heterozygous TOMM40 knockdown mice with ECG alteration; Upregulated Tom40 associated with heat stress-induced cardiomyocyte apoptosis; Reduced expression of Tom40 in hearts of old DCM patients.	(214, 215, 229–238)
Tom5/ TOMM5	TOM complex/ Assisted protein	Lipoprotein-associated phospholipase A2 activity	Correlated with increased activity of lipoprotein-associated phospholipase A2.	(239)
Tim50/ TIMM50	TIM23 complex/ Receptor	Cardiovascular developmental defects; DCM and cardiac hypertrophy	Loss of Tim50 impaired cardiac development in zebrafish embryos; Tim50 deficiency exacerbated cardiac hypertrophy in mice; Downregulated expression of Tim50 in human DCM hearts and in murine hypertrophic hearts.	(240, 241)
Tim23/ TIMM23	TIM23 complex/ Channel	Myocardial H/R or I/R injury; DCM	Reduced Tim23 expression level in hearts of patients with DCM, upon H/R or I/R injury.	(207, 238, 242–244)
mtHSP70/ GRP75	TOM-TIM23 pathway/ PAM	Myocardial H/R injury; Diabetic cardiomyopathy; Chronic AF	Decreased expression of mtHSP70 upon H/R injury, in IFM of T1DM hearts and SSM of T2DM hearts; Increased expression of mtHSP70 in human hearts with chronic AF.	(245–249)
Tim14/ DNAJC19	TOM-TIM23 pathway/ IM cochaperone	DCMA syndrome	Mutations in DNAJC19 were related to DCMA syndrome, a novel autosomal recessive syndrome characterized by early-onset DCM, non-progressive cerebellar ataxia, testicular dysgenesis, growth failure, mild developmental delay, and 3-methylglutaconic aciduria.	(250–255)
MAGMAS/ MAGMAS	TOM-TIM23 pathway/ IM cochaperone	Early death due to heart failure	Two patients from a family with MAGMAS mutation died at 2 years of age of heart failure.	(256)
HSP90/ HSP90A	TOM-TIM23 pathway/ Cytosolic chaperone	Myocardial I/R injury	HSP90 played a beneficial role against myocardial I/R injury.	(208, 217, 257–262)
HSP60/HSPD1 and HSP10/HSPE1	TOM-TIM23 pathway/ MM chaperones	DCM; Myocardial I/R injury; chronic AF; Early death due to heart failure	Cardiac-specific HSP60 deficiency in mice led to DCM; HSP10 overexpression protected against myocardial I/R injury; Both HSP60 and HSP10 were upregulated in human hearts with chronic AF; A girl with HSP60 deficiency died at 2 days of age of heart failure.	(263–266)
MPPα/ PMPCA	TOM-TIM23 pathway/ MPP	PMPCA gene mutation-associated multisystem impairments; Myocardial I/R injury	PMPCA mutants had multisystem impairments, including developmental delay, severe hypotonia, ataxia, lactic acidemia, severe hypertrophic left ventricular cardiomyopathy; Downregulation of MPPα was beneficial to cardiomyocytes during I/R injury.	(258, 267)
MIP/ MIPEP	TOM-TIM23 pathway/ MPP	COXPD31/Eldomery–Sutton syndrome	Mutations in the MIPEP gene caused COXPD31/Eldomery–Sutton syndrome, a recessive disorder with developmental delay, cardiomyopathy, cataracts, hypotonia, left ventricular non-compaction, variable seizures.	(268)
Lon Protease/ LONP1	TOM-TIM23 pathway/ MM Protease	High-fat diet stress; Cardiac hypertrophy; Hypoxia insults; Cardiac aging; Friedreich's ataxia	Lon seemed beneficial to cardiomyocytes upon high-fat diet and hypertrophic stresses but harmful to cardiomyocytes upon hypoxia insults; In aged hearts, Lon expression increased, but proteolytic efficiency declined; In cardiac-specific frataxin-deletion mice, Lon expression and activity were increased in the heart.	(269–276)
YME1L/ YME1L1	TOM-TIM23 pathway/ MM Protease	DCM and heart failure; Experimental autoimmune myocarditis; Myocardial infarction	Cardiac-specific ablation of YME1L in mice led to DCM and heart failure; YME1L was also crucial for the progression of experimental autoimmune myocarditis to DCM and the therapeutic efficacy of mesenchymal stem cells in myocardial infarction.	(195, 277–279)
CLPP/ CLPP	TOM-TIM23 pathway/ MM Protease	DARS2-deficiency–related mitochondrial cardiomyopathy	Loss of CLPP in the heart could alleviate DARS2-deficiency–induced mitochondrial cardiomyopathy.	(280)
AGK/ AGK	TIM22 complex	Sengers syndrome	Mutations in the AGK gene led to Sengers syndrome, an autosomal recessive mitochondrial disorder characterized by hypertrophic cardiomyopathy, congenital cataracts, skeletal myopathy, exercise intolerance, and lactic acidosis.	(281–286)
Tim8a/ TIMM8A	small TIM chaperone in IMS	Myocardial I/R injury	Downregulation of Tim8a was related to the protective role of SB216763 treatment in myocardial I/R stress.	(258)
ALR/ GFER	CHCHD4 Mia40 complex	Cardiac development delay	Inhibition of ALR activity or expression in zebrafish embryos led to retarded cardiac development.	(287)
AIF/ AIFM1	CHCHD4 Mia40 complex	Prenatal ventriculomegaly; Childhood cardiomyopathy; Ischemic cardiomyopathy; DCM	Mutations in the AIFM1 gene led to early prenatal ventriculomegaly and childhood cardiomyopathy; Muscle-specific loss of AIF in mouse led to severe DCM; Harlequin (Hq) mice with AIF deficiency displayed more severe ischemic damage than wild-type hearts.	(288–292)

*I/R, ischemia/reperfusion; mitoBK_Ca_, mitochondrial large conductance voltage and Ca [2+]-dependent K [+] channel; MI, myocardial infarction; DCM, dilated cardiomyopathy; H/R, hypoxia/reoxygenation; AF, atrial fibrillation; IFM, interfibrillar mitochondria; SSM, subsarcolemmal mitochondria; T1DM, type 1 diabetes mellitus; T2DM, type 2 diabetes mellitus; MM, mitochondrial matrix; DNAJC19, DNAJ Homolog, subfamily C, member 19; IM, inner membrane; DCMA, dilated cardiomyopathy with ataxia; MPPα, Mitochondrial processing peptidase subunit alpha; MIP, mitochondrial intermediate peptidase; CLPP, mitochondrial ATP-dependent Clp proteolytic subunit; AGK, acylglycerol kinase; ALR, FAD-linked sulfhydryl oxidase ALR; AIF, apoptosis-inducing factor*.

### Presequence Pathway Associated With Heart Disease

#### TOM Complex in Heart Disease

**Tom20** is an essential receptor subunit of the TOM complex that recognizes mitochondrial precursor proteins with cleavable N-terminal presequences. Tom20 expression was reduced by ischemic insults (206, 207), and showed a cardioprotective role against ischemia/reperfusion (I/R) injury through enhancing the mitochondrial import of PKCepsilon (PKCε) in an HSP90-dependent manner (208). PKCε, a member of the serine/threonine kinase family, has been demonstrated to play a protective role against cardiac I/R injury (208, 216, 293). Additionally, calcium homeostasis, which is closely related to cardiac health, is influenced by Tom20. For example, Wattamon recently reported that the protective role of fibroblast growth factor 2 (FGF-2) against calcium overload was partially mediated by mitochondrial connexin 43 (Cx43) (introduced below), probably in a Tom20-dependent manner (209). Cx43 was imported into mitochondria *via* a Tom20-dependent pathway (257). In another study, Tom20 was reported to be responsible for the direct insertion of VDAC, a protein crucial in regulating cardiac calcium homeostasis (210) through mitochondrial permeability transition pore (mPTP) (294) and mitochondria-associated endoplasmic reticulum membranes (MAMs), into the OM of heart mitochondria in rats (295). Thus, Tom20 could be a crucial adaptor of cardiac calcium homeostasis in a Cx43- or VDAC-associated manner.

**Tom22** serves as the central receptor for both presequence precursors and carrier precursors. Furthermore, it is also the key factor linking the TOM and SAM complex to a supercomplex by interaction with Sam37, which promotes the efficient transfer of β-barrel precursors. Tom22 was recently identified as a potential receptor for cardiac mitochondrial large conductance voltage and Ca^2+^-dependent K [+] (mitoBK_Ca_) channels, facilitating the import of mitoBK_Ca_ *via* the presequence pathway (211). Tom22 deficiency might induce cardiomyocyte dysfunction by interfering with cardiac mitochondrial Ca^2+^ import (211). Additionally, Tom22 has been reported as a potential regulator of heart function through assisting the synthesis of cardiac aldosterone in mitochondria (212). Finally, in chronically hypoxic rat hearts, the level of Tom22 mRNA was increased in cardiac ventricles (213), suggesting a potential role of Tom22 in ischemic heart disease.

**Tom40**, encoded by the TOMM40 gene, forms the main protein-conducting channel of the TOM complex. Previous studies indicated TOMM40 was genetically associated with cardiovascular-related traits (214, 215, 229, 230). Several genome-wide association studies (GWAS) have identified that the TOMM40/APOE locus was strongly associated with low-density lipoprotein cholesterol [rs157580 (231)], high-density lipoprotein cholesterol [rs2075650, (232), rs157581 (233)], high-sensitivity C-reactive protein [rs2075650 (234)], type 2 diabetes [rs157580 (231)], or metabolic syndrome (235). Additionally, experimental evidence showed that homozygous deletion of TOMM40 in mammals was lethal, and heterozygous TOMM40 knockdown mice were found to have cardiac arrhythmia that deteriorated with age (236). Upregulated Tom40 transcription was reported associated with heat stress-induced apoptosis of rat cardiomyocytes (237). Moreover, expression of Tom40 and Tim23 was reduced only in older dilated cardiomyopathy (DCM) patients but not in younger DCM patients, suggesting age-related alterations of these proteins (238).

**Small Tom Proteins (Tom5, Tom6, and Tom7)**. An allele of TOMM5 (the gene encoding Tom5) intronic variant (rs57578064) was correlated with a significant increase in lipoprotein-associated phospholipase A2 activity, which is associated with increased risk of cardiovascular events (239). Tom7 deficit in endothelial cells particularly damaged formation of the cerebrovascular network, but not cardiac vasculature, in zebrafish and mice (296).

#### TIM23 Complex in Heart Disease

**Tim23**, the channel-forming protein of the TIM23 complex, responsible for the translocation of presequence precursors into mitochondrial matrix or IM, was reduced by hypoxia/reoxygenation (H/R) or I/R (207, 242, 243). Restoring expression of Tim23 by various treatments seemed protective (207, 242, 243) against H/R or I/R injuries. However, a controversial study from Bian showed that the protective role of zinc against I/R injury was mediated by enhanced mitophagy, accompanied by downregulation of Tom20 and Tim23 expression (244). In addition, another study reported the association of decreased Tim23 expression in patients with DCM (238).

**Tim50** is the receptor of the TIM23 complex that recognizes presequence carrying proteins. Guo et al. demonstrated that the loss of Tim50 during early zebrafish embryonic development caused neurodegeneration, cardiovascular defects (dysmorphic heart, reduced heartbeat, and decreased circulating blood), and impaired motility. These pathological changes might result from increased cell death, which was mediated by mitochondrial membrane permeabilization and acceleration of cytochrome *c* release (240). Tang et al. further identified that Tim50 was downregulated in both human DCM heart and transverse aortic constriction (TAC)-induced murine hypertrophic heart (241). Meanwhile, global Tim50 knockout mice showed more severe cardiac hypertrophy than wild-type mice, which was alleviated by cardiac-specific overexpression of Tim50 *via* reducing ROS accumulation and ASK1 activity (241).

#### The Presequence-Pathway–Associated Chaperones in Heart Disease

**mtHSP70** (also known as GRP75/ mortalin/ PBP74) is an essential ATP-dependent chaperone of the PAM complex. It drives the translocation of preproteins into the matrix in the membrane-bound motor form and exhibits typical chaperone activity to prevent protein misfolding and aggregation in the soluble form. *In vitro* studies, mtHSP70 was identified as a cardioprotective chaperone against H/R-induced oxidative stress (245, 246), potentially *via* increased import of nuclear genome-encoded antioxidant defense proteins, such as DJ-1 (246). Expression of mtHSP70 was significantly decreased in the interfibrillar mitochondria (IFM) of type 1 diabetes mellitus (T1DM) (247) and the subsarcolemmal mitochondria (SSM) of type 2 diabetes mellitus (T2DM) (248). Cardiac-specific mtHSP70 overexpression restored cardiac function and nuclear-encoded mitochondrial protein import, contributing to a beneficial impact on proteome signature and enhanced mitochondrial function during T2DM (248). Further, mtHSP70 expression was increased in myocardial samples from patients with chronic atrial fibrillation, which suggested an adaptive heat shock response to restore cellular homeostasis (249).

**HSP60 and HSP10** are mitochondrial matrix chaperones, playing pivotal roles in implementing protein folding and preventing protein aggregation. Cardiac-specific HSP60 deficiency in mice led to DCM, heart failure, and lethality. Interestingly, the import of preproteins into mitochondria was unaffected by HSP60 deficiency. However, the imported proteins processed by HSP60 underwent further degradation, suggesting lower stability of those proteins (263). HSP10 showed a similar cardioprotective role in I/R-induced myocyte death (264). Both the beneficial roles of HSP60 and HSP10 in cardiomyocytes were related to the preserved function of Complex I and Complex II. Clinical evidence found upregulated expression of both mitochondrial HSP60 and HSP10 in myocardial samples from patients with chronic atrial fibrillation (265). Agsteribbe et al. reported a single case of a girl who had facial dysmorphic features and breathing difficulties upon birth and died at 2 days of age of heart failure (266). The post-mortem examination revealed that the amount of mitochondrial HSP60 was only about 1/5 of the normal level (266).

**Tim14** (encoded by DNAJC19), human homolog to yeast Pam18/Tim14, is a mitochondrial IM co-chaperone of the TIM23 complex. Mutations in DNAJC19 were related to DCM and cerebellar ataxia (DCMA) syndrome, a novel autosomal recessive syndrome characterized by early-onset DCM with conduction defects, non-progressive cerebellar ataxia, testicular dysgenesis, growth failure, mild developmental delay, and 3-methylglutaconic aciduria, with or without sensorineural hearing loss and basal ganglia lesions (250–255). The pathogenic mechanism of DCMA was associated with protein import inefficiency and cardiolipin remodeling. Experimental evidence suggested DNAJC19 forms a complex with prohibitins (PHBs). Furthermore, the loss of PHB/DNAJC19 complexes affected cardiolipin acylation and led to the accumulation of cardiolipin species with altered acyl chains (297).

**MAGMAS** (mitochondria-associated granulocyte macrophage colony stimulating factor signaling molecule), also termed PAM16/Tim16, forms a stable subcomplex with J-protein Pam18 or DnaJC19. It tethers to the TIM23 complex in yeast and humans (298). Cybel et al. reported that two patients from a family with MAGMAS mutation died at 2 years of age of heart failure (256).

**HSP90**, a chaperone mainly located in the cytoplasm, also played a beneficial role against I/R injury (258, 259) through translocation of PKCε, (208, 217) Cx43, (257, 260) AKT, (261), and Pim1/Lon (262) into mitochondria, potentially *via* the HSP-TOM mitochondrial import pathway.

#### The Presequence-Pathway–Associated Proteinases and Peptidases in Heart Disease

**Mitochondrial Lon Protease** is crucial for the clearance of oxidized or misfolded proteins in the matrix. It played a beneficial role in improving cardiac metabolic flexibility *via* degradation of pyruvate dehydrogenase kinase 4 in mice fed a high-fat diet (269). Moreover, it was also verified to enhance cardiac function *via* improving mitochondrial respiration capacity in pressure overload-induced heart failure in mice (270). However, it seemed harmful to cardiomyocytes upon hypoxia insults, which was associated with enhanced ROS production (271, 272) and accelerated degradation of phosphorylated complex IV subunits (273). In murine heart, mitochondrial Lon protease levels rose with age, but proteolytic efficiency and adaptation to stress were compromised in older animals (274, 275). Mitochondrial Lon protease was also found to be involved in Friedreich's ataxia (FRDA), a rare hereditary neurodegenerative disease characterized by progressive ataxia and cardiomyopathy due to mitochondrial frataxin defect. In cardiac-specific frataxin-deletion mice, a progressive increase in mitochondrial Lon and ClpP protease expression and activity were found in the heart, accompanied by the loss of mitochondrial Fe–S proteins (276).

**YME1L**. As we mentioned above, the balance between long OPA1 (L-OPA1) and short OPA1 (S-OPA1), which is crucial for mitochondrial fusion and fission, is modulated by two mitochondrial proteases, OMA1 and the AAA+ protease YME1L. Cardiac-specific ablation of YME1L in mice led to DCM and heart failure *via* activated OMA1 and accelerated OPA1 proteolysis, which triggered mitochondrial fragmentation and altered cardiac metabolism (195). Moreover, cardiac function and mitochondrial morphology were rescued by Oma1 deletion by preventing OPA1 cleavage (195). The regulation of YME1L in mitochondrial fusion *via* OPA1 proteolysis was further verified in experimental autoimmune myocarditis animals (277) and YME1L-overexpressing or deficit cells (278). Furthermore, it was related to the therapeutic efficacy of mesenchymal stem cells for myocardial infarction (279).

**MPPα**. MPP is a dimeric protease in the matrix that removes N-terminal presequences and consists of MPPα and MPPβ. Mugdha reported that a patient with mutations in the PMPCA gene, which encodes MPPα, had multisystem impairments, including developmental delay, severe hypotonia, ataxia, lactic acidemia, and severe hypertrophic left ventricular cardiomyopathy and died at 14 months from respiratory failure (267). This phenotype may be related to reduced MPPα levels and impaired processing of frataxin and other mitochondrial proteins. Downregulation of MPPα was found linked to the protective role of GSK inhibitor SB216763 in I/R injury (258).

**MIP/Oct1**. Upon removal of the presequence by MPP, some mitochondrial precursor proteins undergo secondary processing carried out by the mitochondrial intermediate peptidase MIP/Oct1 or intermediate cleaving peptidase Icp55/XPNPEP3 to remove destabilizing N-terminal amino acid residues of the imported proteins. Mutations in the MIPEP gene, which encodes MIP, causes COXPD31/Eldomery–Sutton syndrome with developmental delay, cardiomyopathy, left ventricular non-compaction, hypotonia, and infantile death (268).

**CLPP** (mitochondrial ATP-dependent Clp proteolytic subunit), a mitochondrial matrix proteinase, has a central role in protein homeostasis. Loss of CLPP in the heart was found to alleviate mitochondrial cardiomyopathy induced by DARS2 deficiency, potentially mediated by increased *de novo* synthesis of individual OXPHOS subunits, without affecting the mammalian UPR [mt] (280).

### Carrier Pathway Involved in Heart Disease

**Tom70** mainly serves as the receptor for hydrophobic precursors without a cleavable presequence, such as carrier precursors. Tom70 protein was downregulated in hypertrophic heart of animals and humans, which was associated with increased oxidative stress. Furthermore, upregulation of Tom70 provided cardiomyocytes with full resistance to diverse pro-hypertrophic insults (218). Tom70 expression was also reduced by I/R insult in cardiomyocytes (219–222). Supplementation of Tom70 significantly attenuated I/R injury by promoting translocation of PKCε (216, 217) [to increase the expression of KATP channel pore-forming subunit Kir6.2 (223), augment mitochondrial respiratory capacity, and modulate cardiac glucose metabolism (224)], MICU1 (to reduce mitochondrial Ca^2+^ overload), (222) and PINK1 (associated with mitophagy) (221, 225) into mitochondria. Increased expression of PGC-1α/Tom70 was also involved in melatonin-induced cardiac protection against post-myocardial infarction, which was associated with inhibited mitochondrial impairment and reduced ROS generation (219, 220). In the hearts of diabetic db/db mice, Tom70 expression was suppressed. Reconstitution of Tom70 protected against diabetic cardiomyopathy through its antioxidant and antiapoptotic properties (226). Moreover, in aging hearts of diabetic db/db mice, only the expression of mitochondrial membrane proteins like Tom70 and VDAC, but not respiratory enzymes, could be increased by short-term exercise (227). Further, phosphoproteome mapping in a rat model of heart failure revealed phosphorylation of several import machinery proteins (Tom70, HSP90, and Tim8a), suggesting that the modification of mitochondrial protein import was involved in heart failure (228).

**AGK** (acylglycerol kinase). AGK is a mitochondrial lipid kinase that was recently identified as a subunit of the TIM22 complex. It plays an indispensable role in the import and assembly of mitochondrial carrier proteins in the IM (299). It has been shown that loss-of-function mutations in the AGK gene cause Sengers syndrome (281–286), an autosomal recessive mitochondrial disorder characterized by hypertrophic cardiomyopathy, congenital cataracts, skeletal myopathy, exercise intolerance, and lactic acidosis. Loss of AGK led to destabilized TIM22 complex; defects in the biogenesis of carrier substrates (such as adenine nucleotide translocator); lower complex I, III, and IV activities; perturbed tricarboxylic acid (TCA) cycle; and higher citrate synthase activity (300).

**Tim8a** (Tim8a/DDP1 and Tim8b/DDP2 are human homologs of yeast Tim8) is a small TIM chaperone in the IMS. Tim8a expression in ischemic rat heart was downregulated by treatment with GSK inhibitor SB216763, which showed a protective effect against I/R stress (258), suggesting a potential role of Tim8a in ischemic heart disease.

### MIA Machinery Related to Heart Disease

**FAD-linked sulfhydryl oxidase ALR** is the human homolog of yeast Erv1. The interaction between Mia40 and Erv1/ALR facilitates the import of the small Tim proteins and cysteine-rich proteins. Inhibition of ALR activity by MitoBloCK-6 or a translation initiation codon (ATG) morpholino targeted to ALR in zebrafish embryos led to retarded cardiac development and impaired cardiac function (287).

**Apoptosis-Inducing Factor (AIF)** was initially characterized as a pro-apoptotic factor. It translocates from the mitochondrial IMS to the nucleus in the presence of apoptotic insults. It is also critical for the mitochondrial import and maturation of CHCHD4 (in human)/Mia40 (in yeast) (301–303). Mutations of the AIF-encoding gene AIFM1 led to early prenatal ventriculomegaly (288) and childhood cardiomyopathy (289), accompanied by respiratory chain complex I and IV deficiency. Global loss of AIF in mice during embryogenesis resulted in embryonic growth retardation and death during mid-gestation. Muscle-specific loss of AIF in mice led to severe DCM and skeletal muscle atrophy, associated with a significant defect in respiratory chain complex I activity (290). The Harlequin (Hq) mice, a genetic model with an 80% reduction in mitochondrial AIF, displayed more severe ischemic damage than wild-type hearts after acute I/R injury (291, 292).

### Other Molecules Involved in Heart Disease by Impacting Mitochondrial Protein Import Efficiency or Altering Mitochondrial Protein Translocation

**Cardiolipin (CL)** is a unique phospholipid that is localized and synthesized in mitochondrial IM. CL plays a central role in many biological processes, such as mitochondrial biogenesis, protein import, morphology and mitophagy, oxidative phosphorylation, and apoptosis (304–306). Defective remodeling of CL due to genetic mutations of TAZ-1 causes Barth syndrome, a rare, X-linked recessive, infantile-onset debilitating disorder characterized by early-onset cardiomyopathy, skeletal myopathy, growth delay, and neutropenia (304–309). The molecular mechanisms were partially mediated by impaired mitochondrial import machinery. CL was verified to play a central role in the structural integrity and functions of mitochondrial translocases, such as TOM Complex (44, 310), TIM22 Complex, and TIM23 Complex (26, 311, 312).

**Connexin 43 (Cx43)** is the predominant protein forming gap junctions and non-junctional hemichannels in ventricular cardiomyocytes and is also localized at the IM of cardiomyocyte mitochondria (313–315). The translocation of Cx43 to the IM was TOM-HSP90–dependent and was enhanced by ischemic preconditioning (IP). The beneficial role of mitochondrial Cx43 in I/R stress was associated with its regulation of mitochondrial potassium influx and ROS production (257, 313–315). The cardioprotection of IP was abolished by genetic ablation of Cx43, blockade of mitochondrial Cx43 import, or age-related loss of mitochondrial Cx43 (257, 316–318).

**PINK1** is imported into the mitochondrial matrix in healthy conditions, followed by retrotranslocation into cytosol and degradation by the proteasome. Perturbation of this process causes mitophagy, which plays a vital role in the quality control of mitochondria in heart disease. Given that it has been well-studied and summarized in many other reviews (319, 320), we do not discuss it here in detail.

**NDUFB10**, an accessory subunit of complex I, is a substrate of the MIA machinery (CHCHD4/ Mia40) for oxidation-dependent protein import into the mitochondrial IMS. Mutation of cysteine 107 of NDUFB10 impaired its mitochondrial import *via* CHCHD4 and resulted in complex I assembly defect, led to fatal infantile lactic acidosis and cardiomyopathy in a single-case report of an infant (321).

In addition, some other proteins also showed a protective effect against I/R or H/R stress through enhancing their translocation from the cytosol to the mitochondria; these include α-crystallin B (cryAB, the major small heat shock protein in cardiomyocytes) *via* VDAC-Tom20 (322) and DJ-1 *via* mtHSP70 (246, 323–325).

### Other Conditions That Affect Mitochondrial Protein Import Efficiency in Heart Disease

According to their subcellular spatial arrangement in cardiomyocytes, mitochondria are classified into three groups: subsarcolemmal mitochondria (SSM) existing below the cell membrane, interfibrillar mitochondria (IFM) residing in rows between the myofibrils, and perinuclear mitochondria located at the nuclear poles. Mitochondrial subpopulations vary in structure and function and appear to be influenced disparately in different cardiac pathologies, including I/R, heart failure, aging, exercise, and diabetes mellitus. According to recent studies, the mitochondrial import machinery in IFM of T1DM hearts (247, 326) and SSM of T2DM hearts (248, 327) were more susceptible to inefficiency. Further, many studies reported the downregulation of mitochondrial import-machinery components in heart disease, such as heart failure, DCM, ischemic cardiomyopathy, diabetic cardiomyopathy, etc. Supplements of corresponding components could partially recover cardiac function.

However, some studies pointed out an enhancement of mitochondrial protein import in aging animals. Craig et al. found mitochondria from senescent animals exhibited a higher import rate of precursors into the matrix than mitochondria from young animals (328). Later studies showed that, although expression of some key import machinery components was upregulated in the aging heart, import efficiency was compromised (238). The mechanism and significance need to be determined in future studies.

In addition, the import rate of matrix-localized proteins was found to be increased in heart of hyperthyroid animals or by T3 treatment (329–332), which was partially mediated by elevated levels of the OM receptor Tom20 and mtHSP70. Meanwhile, the proteolysis of matrix proteins was unaffected.

## Conclusion

Mitochondrial import machinery pathways are involved in various heart diseases, including heart failure, DCM, hypertrophic cardiomyopathy, ischemic cardiomyopathy, and diabetic cardiomyopathy. Mutants of genes encoding components of the mitochondrial import machinery in humans or genetic deficiency of those genes in animals usually cause severe mitochondrial cardiomyopathy and are lethal, highlighting the critical role of mitochondrial import machinery in heart disease. However, compared with neurodegenerative diseases, in which the functions of mitochondrial import machinery are relatively well-studied and established, research in heart disease is still fairly limited. Although many studies detected some components of mitochondrial import machinery, most studies simply regarded those components as indicators of mitochondrial content to evaluate mitochondrial biogenesis or mitophagy. The function of mitochondrial import machinery was highly neglected. Actually, even from the perspective of mitochondrial biogenesis or mitophagy, under different stimuli, import machinery components are able to adapt to diverse cellular functions, which are not always proportional to mitochondrial quantity.

Furthermore, our current understanding of mitochondrial import machinery in heart disease is still widely based on experimental evidence from yeast. Nevertheless, recent research in higher eukaryotes has identified more complex and diverse functions in some conserved components of mitochondrial import machinery. Furthermore, with the development of high-throughput sequencing in genomics, transcriptomics, proteomics, and metabolomics, more and more novel import machinery components have been revealed in mammals. The roles of mitochondrial import machinery in heart disease deserve considerable attention, and future studies are urgently needed.

## Author Contributions

FZ devised the original idea of the work, searched the literature, and wrote the manuscript. M-HZ edited the manuscript. All authors made significant contributions to this work and approved it for publication.

## Funding

M-HZ's lab was supported by funding from the following agencies: National Heart, Lung, and Blood Institute (HL079584, HL080499, HL089920, HL110488, HL128014, HL132500, HL137371, and HL142287) and National Cancer Institute (CA213022). M-HZ is the Eminent Scholar in Molecular and Translational Medicine of the Georgia Research Alliance.

## Conflict of Interest

The authors declare that the research was conducted in the absence of any commercial or financial relationships that could be construed as a potential conflict of interest.

## Publisher's Note

All claims expressed in this article are solely those of the authors and do not necessarily represent those of their affiliated organizations, or those of the publisher, the editors and the reviewers. Any product that may be evaluated in this article, or claim that may be made by its manufacturer, is not guaranteed or endorsed by the publisher.

## References

[1] Dennerlein S Wang C Rehling P. Plasticity of mitochondrial translation. Trends Cell Biol. (2017) 27:712–21. 10.1016/j.tcb.2017.05.004 28606446

[2] Schmidt O Harbauer AB Rao S Eyrich B Zahedi RP Stojanovski D. Regulation of mitochondrial protein import by cytosolic kinases. Cell. (2011) 144:227–39. 10.1016/j.cell.2010.12.015 21215441

[3] Wiedemann N Pfanner N. Mitochondrial machineries for protein import and assembly. Annu Rev Biochem. (2017) 86:685–714. 10.1146/annurev-biochem-060815-014352 28301740

[4] Pfanner N Warscheid B Wiedemann N. Mitochondrial proteins: from biogenesis to functional networks. Nat Rev Mol Cell Biol. (2019) 20:267–84. 10.1038/s41580-018-0092-0 30626975 PMC6684368

[5] Nicolas E Tricarico R Savage M Golemis EA Hall MJ. Disease-associated genetic variation in human mitochondrial protein import. Am J Hum Genet. (2019) 104:784–801. 10.1016/j.ajhg.2019.03.019 31051112 PMC6506819

[6] Jackson TD Palmer CS Stojanovski D. Mitochondrial diseases caused by dysfunctional mitochondrial protein import. Biochem Soc Trans. (2018) 46:1225–38. 10.1042/BST20180239 30287509

[7] Vogtle FN Wortelkamp S Zahedi RP Becker D Leidhold C Gevaert K. Global analysis of the mitochondrial N-proteome identifies a processing peptidase critical for protein stability. Cell. (2009) 139:428–39. 10.1016/j.cell.2009.07.045 19837041

[8] Roise D Horvath SJ Tomich JM Richards JH Schatz G. A chemically synthesized pre-sequence of an imported mitochondrial protein can form an amphiphilic helix and perturb natural and artificial phospholipid bilayers. EMBO J. (1986) 5:1327–34. 10.1002/j.1460-2075.1986.tb04363.x 3015598 PMC1166944

[9] Abe Y Shodai T Muto T Mihara K Torii H Nishikawa S. Structural basis of presequence recognition by the mitochondrial protein import receptor Tom20. Cell. (2000) 100:551–60. 10.1016/S0092-8674(00)80691-1 10721992

[10] van Wilpe S Ryan MT Hill K Maarse AC Meisinger C Brix J. Tom22 is a multifunctional organizer of the mitochondrial preprotein translocase. Nature. (1999) 401:485–9. 10.1038/46802 10519552

[11] Mokranjac D Neupert W. Cell biology: architecture of a protein entry gate. Nature. (2015) 528:201–2. 10.1038/nature16318 26605527

[12] Lohret TA Jensen RE Kinnally KW. Tim23, a protein import component of the mitochondrial inner membrane, is required for normal activity of the multiple conductance channel, MCC. J Cell Biol. (1997) 137:377–86. 10.1083/jcb.137.2.377 9128249 PMC2139772

[13] Bauer MF Sirrenberg C Neupert W Brunner M. Role of Tim23 as voltage sensor and presequence receptor in protein import into mitochondria. Cell. (1996) 87:33–41. 10.1016/S0092-8674(00)81320-3 8858146

[14] Dekker PJ Keil P Rassow J Maarse AC Pfanner N Meijer M. Identification of MIM23, a putative component of the protein import machinery of the mitochondrial inner membrane. FEBS Lett. (1993) 330:66–70. 10.1016/0014-5793(93)80921-G 8370462

[15] Demishtein-Zohary K Marom M Neupert W Mokranjac D Azem A. GxxxG motifs hold the TIM23 complex together. FEBS J. (2015) 282:2178–86. 10.1111/febs.13266 25765297

[16] Chacinska A Lind M Frazier AE Dudek J Meisinger C Geissler A. Mitochondrial presequence translocase: switching between TOM tethering and motor recruitment involves Tim21 and Tim17. Cell. (2005) 120:817–29. 10.1016/j.cell.2005.01.011 15797382

[17] Kang PJ Ostermann J Shilling J Neupert W Craig EA Pfanner N. Requirement for hsp70 in the mitochondrial matrix for translocation and folding of precursor proteins. Nature. (1990) 348:137–43. 10.1038/348137a0 2234077

[18] Horst M Oppliger W Rospert S Schonfeld HJ Schatz G Azem A. Sequential action of two hsp70 complexes during protein import into mitochondria. EMBO J. (1997) 16:1842–9. 10.1093/emboj/16.8.1842 9155010 PMC1169787

[19] Demishtein-Zohary K Gunsel U Marom M Banerjee R Neupert W Azem A. Role of Tim17 in coupling the import motor to the translocation channel of the mitochondrial presequence translocase. Elife. (2017) 6:22696. 10.7554/eLife.22696 28165323 PMC5308891

[20] Ting SY Yan NL Schilke BA Craig EA. Dual interaction of scaffold protein Tim44 of mitochondrial import motor with channel-forming translocase subunit Tim23. Elife. (2017) 6:23609. 10.7554/eLife.23609 28440746 PMC5422074

[21] Banerjee R Gladkova C Mapa K Witte G Mokranjac D. Protein translocation channel of mitochondrial inner membrane and matrix-exposed import motor communicate via two-domain coupling protein. Elife. (2015) 4:e11897. 10.7554/eLife.11897 26714107 PMC4749553

[22] Sikor M Mapa K von Voithenberg LV Mokranjac D Lamb DC. Real-time observation of the conformational dynamics of mitochondrial Hsp70 by spFRET. EMBO J. (2013) 32:1639–49. 10.1038/emboj.2013.89 23624933 PMC3671257

[23] Schendzielorz AB Schulz C Lytovchenko O Clancy A Guiard B Ieva R. Two distinct membrane potential-dependent steps drive mitochondrial matrix protein translocation. J Cell Biol. (2017) 216:83–92. 10.1083/jcb.201607066 28011846 PMC5223606

[24] Schulz C Rehling P. Remodelling of the active presequence translocase drives motor-dependent mitochondrial protein translocation. Nat Commun. (2014) 5:4349. 10.1038/ncomms5349 25008211

[25] Truscott KN Kovermann P Geissler A Merlin A Meijer M Driessen AJ. A presequence- and voltage-sensitive channel of the mitochondrial preprotein translocase formed by Tim23. Nat Struct Biol. (2001) 8:1074–82. 10.1038/nsb726 11713477

[26] Malhotra K Modak A Nangia S Daman TH Gunsel U Robinson VL. Cardiolipin mediates membrane and channel interactions of the mitochondrial TIM23 protein import complex receptor Tim50. Sci Adv. (2017) 3:e1700532. 10.1126/sciadv.1700532 28879236 PMC5580885

[27] Denkert N Schendzielorz AB Barbot M Versemann L Richter F Rehling P. Cation selectivity of the presequence translocase channel Tim23 is crucial for efficient protein import. Elife. (2017) 6:28324. 10.7554/eLife.28324 28857742 PMC5578737

[28] Ramesh A Peleh V Martinez-Caballero S Wollweber F Sommer F van der Laan M. A disulfide bond in the TIM23 complex is crucial for voltage gating and mitochondrial protein import. J Cell Biol. (2016) 214:417–31. 10.1083/jcb.201602074 27502485 PMC4987294

[29] Hawlitschek G Schneider H Schmidt B Tropschug M Hartl FU Neupert W. Mitochondrial protein import: identification of processing peptidase and of PEP, a processing enhancing protein. Cell. (1988) 53:795–806. 10.1016/0092-8674(88)90096-7 2967109

[30] Neupert W. A perspective on transport of proteins into mitochondria: a myriad of open questions. J Mol Biol. (2015) 427:1135–58. 10.1016/j.jmb.2015.02.001 25676309

[31] Fukasawa Y Tsuji J Fu SC Tomii K Horton P Imai K. MitoFates: improved prediction of mitochondrial targeting sequences and their cleavage sites. Mol Cell Proteomics. (2015) 14:1113–26. 10.1074/mcp.M114.043083 25670805 PMC4390256

[32] Varshavsky A. The N-end rule pathway and regulation by proteolysis. Protein Sci. (2011) 20:1298–345. 10.1002/pro.666 21633985 PMC3189519

[33] Veling MT Reidenbach AG Freiberger EC Kwiecien NW Hutchins PD Drahnak MJ. Multi-omic mitoprotease profiling defines a role for Oct1p in coenzyme Q production. Mol Cell. (2017) 68:970–77 e11. 10.1016/j.molcel.2017.11.023 29220658 PMC5730362

[34] Vogtle FN Prinz C Kellermann J Lottspeich F Pfanner N Meisinger C. Mitochondrial protein turnover: role of the precursor intermediate peptidase Oct1 in protein stabilization. Mol Biol Cell. (2011) 22:2135–43. 10.1091/mbc.e11-02-0169 21525245 PMC3128517

[35] Ieva R Schrempp SG Opalinski L Wollweber F Hoss P Heisswolf AK. Mgr2 functions as lateral gatekeeper for preprotein sorting in the mitochondrial inner membrane. Mol Cell. (2014) 56:641–52. 10.1016/j.molcel.2014.10.010 25454944

[36] Matta SK Kumar A D'Silva P. Mgr2 regulates mitochondrial preprotein import by associating with channel-forming Tim23 subunit. Mol Biol Cell. (2020) 31:1112–23. 10.1091/mbc.E19-12-0677 32186971 PMC7353164

[37] Schendzielorz AB Bragoszewski P Naumenko N Gomkale R Schulz C Guiard B. Motor recruitment to the TIM23 channel's lateral gate restricts polypeptide release into the inner membrane. Nat Commun. (2018) 9:4028. 10.1038/s41467-018-06492-8 30279421 PMC6168564

[38] Stiller SB Hopker J Oeljeklaus S Schutze C Schrempp SG Vent-Schmidt J. Mitochondrial OXA translocase plays a major role in biogenesis of inner-membrane proteins. Cell Metab. (2016) 23:901–8. 10.1016/j.cmet.2016.04.005 27166948 PMC4873616

[39] Park K Botelho SC Hong J Osterberg M Kim H. Dissecting stop transfer vs. conservative sorting pathways for mitochondrial inner membrane proteins in vivo. J Biol Chem. (2013) 288:1521–32. 10.1074/jbc.M112.409748 23184936 PMC3548465

[40] Bohnert M Rehling P Guiard B Herrmann JM Pfanner N van der Laan M. Cooperation of stop-transfer and conservative sorting mechanisms in mitochondrial protein transport. Curr Biol. (2010) 20:1227–32. 10.1016/j.cub.2010.05.058 20619821

[41] Herrmann JM Neupert W Stuart RA. Insertion into the mitochondrial inner membrane of a polytopic protein, the nuclear-encoded Oxa1p. EMBO J. (1997) 16:2217–26. 10.1093/emboj/16.9.2217 9171337 PMC1169824

[42] Hell K Neupert W Stuart RA. Oxa1p acts as a general membrane insertion machinery for proteins encoded by mitochondrial DNA. EMBO J. (2001) 20:1281–8. 10.1093/emboj/20.6.1281 11250894 PMC145526

[43] Kiebler M Pfaller R Sollner T Griffiths G Horstmann H Pfanner N. Identification of a mitochondrial receptor complex required for recognition and membrane insertion of precursor proteins. Nature. (1990) 348:610–6. 10.1038/348610a0 2174514

[44] Hill K Model K Ryan MT Dietmeier K Martin F Wagner R. Tom40 forms the hydrophilic channel of the mitochondrial import pore for preproteins [see comment]. Nature. (1998) 395:516–21. 10.1038/26780 9774109

[45] Shiota T Imai K Qiu J Hewitt VL Tan K Shen HH. Molecular architecture of the active mitochondrial protein gate. Science. (2015) 349:1544–8. 10.1126/science.aac6428 26404837

[46] Yamano K Yatsukawa Y Esaki M Hobbs AE Jensen RE Endo T. Tom20 and Tom22 share the common signal recognition pathway in mitochondrial protein import. J Biol Chem. (2008) 283:3799–807. 10.1074/jbc.M708339200 18063580

[47] Young JC Hoogenraad NJ Hartl FU. Molecular chaperones Hsp90 and Hsp70 deliver preproteins to the mitochondrial import receptor Tom70. Cell. (2003) 112:41–50. 10.1016/S0092-8674(02)01250-3 12526792

[48] Bhangoo MK Tzankov S Fan AC Dejgaard K Thomas DY Young JC. Multiple 40-kDa heat-shock protein chaperones function in Tom70-dependent mitochondrial import. Mol Biol Cell. (2007) 18:3414–28. 10.1091/mbc.e07-01-0088 17596514 PMC1951752

[49] Wu Y Sha B. Crystal structure of yeast mitochondrial outer membrane translocon member Tom70p. Nat Struct Mol Biol. (2006) 13:589–93. 10.1038/nsmb1106 16767096

[50] Backes S Hess S Boos F Woellhaf MW Godel S Jung M. Tom70 enhances mitochondrial preprotein import efficiency by binding to internal targeting sequences. J Cell Biol. (2018) 217:1369–82. 10.1083/jcb.201708044 29382700 PMC5881500

[51] Melin J Kilisch M Neumann P Lytovchenko O Gomkale R Schendzielorz A. A presequence-binding groove in Tom70 supports import of Mdl1 into mitochondria. Biochim Biophys Acta. (2015) 1853:1850–9. 10.1016/j.bbamcr.2015.04.021 25958336

[52] Yamamoto H Fukui K Takahashi H Kitamura S Shiota T Terao K. Roles of Tom70 in import of presequence-containing mitochondrial proteins. J Biol Chem. (2009) 284:31635–46. 10.1074/jbc.M109.041756 19767391 PMC2797234

[53] Bayrhuber M Meins T Habeck M Becker S Giller K Villinger S. Structure of the human voltage-dependent anion channel. Proc Natl Acad Sci USA. (2008) 105:15370–5. 10.1073/pnas.0808115105 18832158 PMC2557026

[54] Qiu J Wenz LS Zerbes RM Oeljeklaus S Bohnert M Stroud DA. Coupling of mitochondrial import and export translocases by receptor-mediated supercomplex formation. Cell. (2013) 154:596–608. 10.1016/j.cell.2013.06.033 23911324

[55] Lackey SW Taylor RD Go NE Wong A Sherman EL Nargang FE. Evidence supporting the 19 beta-strand model for Tom40 from cysteine scanning and protease site accessibility studies. J Biol Chem. (2014) 289:21640–50. 10.1074/jbc.M114.578765 24947507 PMC4118123

[56] Esaki M Kanamori T Nishikawa S Shin I Schultz PG Endo T. Tom40 protein import channel binds to non-native proteins and prevents their aggregation. Nat Struct Biol. (2003) 10:988–94. 10.1038/nsb1008 14595396

[57] Melin J Schulz C Wrobel L Bernhard O Chacinska A Jahn O. Presequence recognition by the tom40 channel contributes to precursor translocation into the mitochondrial matrix. Mol Cell Biol. (2014) 34:3473–85. 10.1128/MCB.00433-14 25002531 PMC4135617

[58] Yamano K Tanaka-Yamano S Endo T. Tom7 regulates Mdm10-mediated assembly of the mitochondrial import channel protein Tom40. J Biol Chem. (2010) 285:41222–31. 10.1074/jbc.M110.163238 21036907 PMC3009848

[59] Dietmeier K Honlinger A Bomer U Dekker PJ Eckerskorn C Lottspeich F. Tom5 functionally links mitochondrial preprotein receptors to the general import pore. Nature. (1997) 388:195–200. 10.1038/40663 9217162

[60] Gornicka A Bragoszewski P Chroscicki P Wenz LS Schulz C Rehling P. discrete pathway for the transfer of intermembrane space proteins across the outer membrane of mitochondria. Mol Biol Cell. (2014) 25:3999–4009. 10.1091/mbc.e14-06-1155 25318675 PMC4263444

[61] Kurz M Martin H Rassow J Pfanner N Ryan MT. Biogenesis of Tim proteins of the mitochondrial carrier import pathway: differential targeting mechanisms and crossing over with the main import pathway. Mol Biol Cell. (1999) 10:2461–74. 10.1091/mbc.10.7.2461 10397776 PMC25469

[62] Schulz C Schendzielorz A Rehling P. Unlocking the presequence import pathway. Trends Cell Biol. (2015) 25:265–75. 10.1016/j.tcb.2014.12.001 25542066

[63] Rahman B Kawano S Yunoki-Esaki K Anzai T Endo T NMR. analyses on the interactions of the yeast Tim50 C-terminal region with the presequence and Tim50 core domain. FEBS Lett. (2014) 588:678–84. 10.1016/j.febslet.2013.12.037 24462684

[64] Martinez-Caballero S Grigoriev SM Herrmann JM Campo ML Kinnally KW. Tim17p regulates the twin pore structure and voltage gating of the mitochondrial protein import complex TIM23. J Biol Chem. (2007) 282:3584–93. 10.1074/jbc.M607551200 17148445

[65] van der Laan M Wiedemann N Mick DU Guiard B Rehling P Pfanner N. role for Tim21 in membrane-potential-dependent preprotein sorting in mitochondria. Curr Biol. (2006) 16:2271–6. 10.1016/j.cub.2006.10.025 17113393

[66] Meinecke M Wagner R Kovermann P Guiard B Mick DU Hutu DP. Tim50 maintains the permeability barrier of the mitochondrial inner membrane. Science. (2006) 312:1523–6. 10.1126/science.1127628 16763150

[67] Tamura Y Harada Y Shiota T Yamano K Watanabe K Yokota M. Tim23-Tim50 pair coordinates functions of translocators and motor proteins in mitochondrial protein import. J Cell Biol. (2009) 184:129–41. 10.1083/jcb.200808068 19139266 PMC2615085

[68] Mokranjac D Sichting M Popov-Celeketic D Mapa K Gevorkyan-Airapetov L Zohary K. Role of Tim50 in the transfer of precursor proteins from the outer to the inner membrane of mitochondria. Mol Biol Cell. (2009) 20:1400–7. 10.1091/mbc.e08-09-0934 19144822 PMC2649253

[69] Gevorkyan-Airapetov L Zohary K Popov-Celeketic D Mapa K Hell K Neupert W. Interaction of Tim23 with Tim50 is essential for protein translocation by the mitochondrial TIM23 complex. J Biol Chem. (2009) 284:4865–72. 10.1074/jbc.M807041200 19017642

[70] Rainbolt TK Atanassova N Genereux JC Wiseman RL. Stress-regulated translational attenuation adapts mitochondrial protein import through Tim17A degradation. Cell Metab. (2013) 18:908–19. 10.1016/j.cmet.2013.11.006 24315374 PMC3904643

[71] Wrobel L Sokol AM Chojnacka M Chacinska A. The presence of disulfide bonds reveals an evolutionarily conserved mechanism involved in mitochondrial protein translocase assembly. Sci Rep. (2016) 6:27484. 10.1038/srep27484 27265872 PMC4893733

[72] Lytovchenko O Melin J Schulz C Kilisch M Hutu DP Rehling P. Signal recognition initiates reorganization of the presequence translocase during protein import. EMBO J. (2013) 32:886–98. 10.1038/emboj.2013.23 23403928 PMC3604718

[73] Mokranjac D Popov-Celeketic D Hell K Neupert W. Role of Tim21 in mitochondrial translocation contact sites. J Biol Chem. (2005) 280:23437–40. 10.1074/jbc.C500135200 15878866

[74] Albrecht R Rehling P Chacinska A Brix J Cadamuro SA Volkmer R. The Tim21 binding domain connects the preprotein translocases of both mitochondrial membranes. EMBO Rep. (2006) 7:1233–8. 10.1038/sj.embor.7400828 17099692 PMC1794701

[75] Wiedemann N van der Laan M Hutu DP Rehling P Pfanner N. Sorting switch of mitochondrial presequence translocase involves coupling of motor module to respiratory chain. J Cell Biol. (2007) 179:1115–22. 10.1083/jcb.200709087 18070913 PMC2140023

[76] Gebert M Schrempp SG Mehnert CS Heisswolf AK Oeljeklaus S Ieva R. Mgr2 promotes coupling of the mitochondrial presequence translocase to partner complexes. J Cell Biol. (2012) 197:595–604. 10.1083/jcb.201110047 22613836 PMC3365495

[77] Martin J Mahlke K Pfanner N. Role of an energized inner membrane in mitochondrial protein import. Delta psi drives the movement of presequences. J Biol Chem. (1991) 266:18051–7. 10.1016/S0021-9258(18)55235-2 1833391

[78] Turakhiya U von der Malsburg K Gold VAM Guiard B Chacinska A van der Laan M. Protein import by the mitochondrial presequence translocase in the absence of a membrane potential. J Mol Biol. (2016) 428:1041–52. 10.1016/j.jmb.2016.01.020 26827728

[79] Popov-Celeketic D Mapa K Neupert W Mokranjac D. Active remodelling of the TIM23 complex during translocation of preproteins into mitochondria. EMBO J. (2008) 27:1469–80. 10.1038/emboj.2008.79 18418384 PMC2396396

[80] Ting SY Schilke BA Hayashi M Craig EA. Architecture of the TIM23 inner mitochondrial translocon and interactions with the matrix import motor. J Biol Chem. (2014) 289:28689–96. 10.1074/jbc.M114.588152 25157107 PMC4192517

[81] Dekker PJ Pfanner N. Role of mitochondrial GrpE and phosphate in the ATPase cycle of matrix Hsp70. J Mol Biol. (1997) 270:321–7. 10.1006/jmbi.1997.1131 9237899

[82] Mokranjac D Bourenkov G Hell K Neupert W Groll M. Structure and function of Tim14 and Tim16, the J and J-like components of the mitochondrial protein import motor. EMBO J. (2006) 25:4675–85. 10.1038/sj.emboj.7601334 16977310 PMC1590002

[83] D'Silva PR Schilke B Hayashi M Craig EA. Interaction of the J-protein heterodimer Pam18/Pam16 of the mitochondrial import motor with the translocon of the inner membrane. Mol Biol Cell. (2008) 19:424–32. 10.1091/mbc.e07-08-0748 18003975 PMC2174176

[84] Kozany C Mokranjac D Sichting M Neupert W Hell K. The J domain-related cochaperone Tim16 is a constituent of the mitochondrial TIM23 preprotein translocase. Nat Struct Mol Biol. (2004) 11:234–41. 10.1038/nsmb734 14981506

[85] Frazier AE Dudek J Guiard B Voos W Li Y Lind M. Pam16 has an essential role in the mitochondrial protein import motor. Nat Struct Mol Biol. (2004) 11:226–33. 10.1038/nsmb735 14981507

[86] Li Y Dudek J Guiard B Pfanner N Rehling P Voos W. The presequence translocase-associated protein import motor of mitochondria. Pam16 functions in an antagonistic manner to Pam18. J Biol Chem. (2004) 279:38047–54. 10.1074/jbc.M404319200 15218029

[87] van der Laan M Chacinska A Lind M Perschil I Sickmann A Meyer HE. Pam17 is required for architecture and translocation activity of the mitochondrial protein import motor. Mol Cell Biol. (2005) 25:7449–58. 10.1128/MCB.25.17.7449-7458.2005 16107694 PMC1190294

[88] Taylor AB Smith BS Kitada S Kojima K Miyaura H Otwinowski Z. Crystal structures of mitochondrial processing peptidase reveal the mode for specific cleavage of import signal sequences. Structure. (2001) 9:615–25. 10.1016/S0969-2126(01)00621-9 11470436

[89] Naamati A Regev-Rudzki N Galperin S Lill R Pines O. Dual targeting of Nfs1 and discovery of its novel processing enzyme, Icp55. J Biol Chem. (2009) 284:30200–8. 10.1074/jbc.M109.034694 19720832 PMC2781575

[90] Gakh O Cavadini P Isaya G. Mitochondrial processing peptidases. Biochim Biophys Acta. (2002) 1592:63–77. 10.1016/S0167-4889(02)00265-3 12191769

[91] Koppen M Langer T. Protein degradation within mitochondria: versatile activities of AAA proteases and other peptidases. Crit Rev Biochem Mol Biol. (2007) 42:221–42. 10.1080/10409230701380452 17562452

[92] Ostermann J Horwich AL Neupert W Hartl FU. Protein folding in mitochondria requires complex formation with hsp60 and ATP hydrolysis. Nature. (1989) 341:125–30. 10.1038/341125a0 2528694

[93] Johnson KA Bhushan S Stahl A Hallberg BM Frohn A Glaser E. The closed structure of presequence protease PreP forms a unique 10,000 Angstroms3 chamber for proteolysis. EMBO J. (2006) 25:1977–86. 10.1038/sj.emboj.7601080 16601675 PMC1456932

[94] Mossmann D Vogtle FN Taskin AA Teixeira PF Ring J Burkhart JM. Amyloid-beta peptide induces mitochondrial dysfunction by inhibition of preprotein maturation. Cell Metab. (2014) 20:662–9. 10.1016/j.cmet.2014.07.024 25176146

[95] He S Fox TD. Membrane translocation of mitochondrially coded Cox2p: distinct requirements for export of N and C termini and dependence on the conserved protein Oxa1p. Mol Biol Cell. (1997) 8:1449–60. 10.1091/mbc.8.8.1449 9285818 PMC276169

[96] Pfeffer S Woellhaf MW Herrmann JM Forster F. Organization of the mitochondrial translation machinery studied in situ by cryoelectron tomography. Nat Commun. (2015) 6:6019. 10.1038/ncomms7019 25609543

[97] Ott M Herrmann JM. Co-translational membrane insertion of mitochondrially encoded proteins. Biochim Biophys Acta. (2010) 1803:767–75. 10.1016/j.bbamcr.2009.11.010 19962410

[98] Hell K Herrmann JM Pratje E Neupert W Stuart RA. Oxa1p, an essential component of the N-tail protein export machinery in mitochondria. Proc Natl Acad Sci USA. (1998) 95:2250–5. 10.1073/pnas.95.5.2250 9482871 PMC19309

[99] Hartl FU Schmidt B Wachter E Weiss H Neupert W. Transport into mitochondria and intramitochondrial sorting of the Fe/S protein of ubiquinol-cytochrome c reductase. Cell. (1986) 47:939–51. 10.1016/0092-8674(86)90809-3 3022944

[100] Rojo EE Stuart RA Neupert W. Conservative sorting of F0-ATPase subunit 9: export from matrix requires delta pH across inner membrane and matrix ATP. EMBO J. (1995) 14:3445–51. 10.1002/j.1460-2075.1995.tb07350.x 7628445 PMC394411

[101] Hildenbeutel M Theis M Geier M Haferkamp I Neuhaus HE Herrmann JM. The membrane insertase Oxa1 is required for efficient import of carrier proteins into mitochondria. J Mol Biol. (2012) 423:590–9. 10.1016/j.jmb.2012.07.018 22846909

[102] Hansen KG Herrmann JM. Transport of proteins into mitochondria. Protein J. (2019) 38:330–42. 10.1007/s10930-019-09819-6 30868341

[103] Endres M Neupert W Brunner M. Transport of the ADP/ATP carrier of mitochondria from the TOM complex to the TIM2254 complex. EMBO J. (1999) 18:3214–21. 10.1093/emboj/18.12.3214 10369662 PMC1171402

[104] Brix J Rudiger S Bukau B Schneider-Mergener J Pfanner N. Distribution of binding sequences for the mitochondrial import receptors Tom20, Tom22, and Tom70 in a presequence-carrying preprotein and a non-cleavable preprotein. J Biol Chem. (1999) 274:16522–30. 10.1074/jbc.274.23.16522 10347216

[105] Faou P Hoogenraad NJ. Tom34: a cytosolic cochaperone of the Hsp90/Hsp70 protein complex involved in mitochondrial protein import. Biochim Biophys Acta. (2012) 1823:348–57. 10.1016/j.bbamcr.2011.12.001 22178133

[106] Gava LM Goncalves DC Borges JC Ramos CH. Stoichiometry and thermodynamics of the interaction between the C-terminus of human 90kDa heat shock protein Hsp90 and the mitochondrial translocase of outer membrane Tom70. Arch Biochem Biophys. (2011) 513:119–25. 10.1016/j.abb.2011.06.015 21781956

[107] Baker MJ Webb CT Stroud DA Palmer CS Frazier AE Guiard B. Structural and functional requirements for activity of the Tim9-Tim10 complex in mitochondrial protein import. Mol Biol Cell. (2009) 20:769–79. 10.1091/mbc.e08-09-0903 19037098 PMC2633397

[108] Koehler CM Merchant S Oppliger W Schmid K Jarosch E Dolfini L. Tim9p, an essential partner subunit of Tim10p for the import of mitochondrial carrier proteins. EMBO J. (1998) 17:6477–86. 10.1093/emboj/17.22.6477 9822593 PMC1170995

[109] Sirrenberg C Endres M Folsch H Stuart RA Neupert W Brunner M. Carrier protein import into mitochondria mediated by the intermembrane proteins Tim10/Mrs11 and Tim12/Mrs5. Nature. (1998) 391:912–5. 10.1038/36136 9495346

[110] Weinhaupl K Lindau C Hessel A Wang Y Schutze C Jores T. Structural basis of membrane protein chaperoning through the mitochondrial intermembrane space. Cell. (2018) 175:1365–79 e25. 10.1016/j.cell.2018.10.039 30445040 PMC6242696

[111] Sirrenberg C Bauer MF Guiard B Neupert W Brunner M. Import of carrier proteins into the mitochondrial inner membrane mediated by Tim22. Nature. (1996) 384:582–5. 10.1038/384582a0 8955274

[112] Koehler CM Jarosch E Tokatlidis K Schmid K Schweyen RJ Schatz G. Import of mitochondrial carriers mediated by essential proteins of the intermembrane space. Science. (1998) 279:369–73. 10.1126/science.279.5349.369 9430585

[113] Okamoto H Miyagawa A Shiota T Tamura Y Endo T. Intramolecular disulfide bond of Tim22 protein maintains integrity of the TIM22 complex in the mitochondrial inner membrane. J Biol Chem. (2014) 289:4827–38. 10.1074/jbc.M113.543264 24385427 PMC3931045

[114] Wrobel L Trojanowska A Sztolsztener ME Chacinska A. Mitochondrial protein import: Mia40 facilitates Tim22 translocation into the inner membrane of mitochondria. Mol Biol Cell. (2013) 24:543–54. 10.1091/mbc.e12-09-0649 23283984 PMC3583659

[115] Rehling P Model K Brandner K Kovermann P Sickmann A Meyer HE. Protein insertion into the mitochondrial inner membrane by a twin-pore translocase. Science. (2003) 299:1747–51. 10.1126/science.1080945 12637749

[116] Kerscher O Holder J Srinivasan M Leung RS Jensen RE. The Tim54p-Tim22p complex mediates insertion of proteins into the mitochondrial inner membrane. J Cell Biol. (1997) 139:1663–75. 10.1083/jcb.139.7.1663 9412462 PMC2132641

[117] Li J Qian X Hu J Sha B. Molecular chaperone Hsp70/Hsp90 prepares the mitochondrial outer membrane translocon receptor Tom71 for preprotein loading. J Biol Chem. (2009) 284:23852–9. 10.1074/jbc.M109.023986 19581297 PMC2749157

[118] Wiedemann N Pfanner N Ryan MT. The three modules of ADP/ATP carrier cooperate in receptor recruitment and translocation into mitochondria. EMBO J. (2001) 20:951–60. 10.1093/emboj/20.5.951 11230119 PMC145466

[119] Curran SP Leuenberger D Schmidt E Koehler CM. The role of the Tim8p-Tim13p complex in a conserved import pathway for mitochondrial polytopic inner membrane proteins. J Cell Biol. (2002) 158:1017–27. 10.1083/jcb.200205124 12221072 PMC2173223

[120] Webb CT Gorman MA Lazarou M Ryan MT Gulbis JM. Crystal structure of the mitochondrial chaperone TIM910 reveals a six-bladed alpha-propeller. Mol Cell. (2006) 21:123–33. 10.1016/j.molcel.2005.11.010 16387659

[121] Curran SP Leuenberger D Oppliger W Koehler CM. The Tim9p-Tim10p complex binds to the transmembrane domains of the ADP/ATP carrier. EMBO J. (2002) 21:942–53. 10.1093/emboj/21.5.942 11867522 PMC125908

[122] Davis AJ Alder NN Jensen RE Johnson AE. The Tim9p/10p and Tim8p/13p complexes bind to specific sites on Tim23p during mitochondrial protein import. Mol Biol Cell. (2007) 18:475–86. 10.1091/mbc.e06-06-0546 17122363 PMC1783793

[123] Gebert N Chacinska A Wagner K Guiard B Koehler CM Rehling P. Assembly of the three small Tim proteins precedes docking to the mitochondrial carrier translocase. EMBO Rep. (2008) 9:548–54. 10.1038/embor.2008.49 18421298 PMC2427372

[124] Wagner K Gebert N Guiard B Brandner K Truscott KN Wiedemann N. The assembly pathway of the mitochondrial carrier translocase involves four preprotein translocases. Mol Cell Biol. (2008) 28:4251–60. 10.1128/MCB.02216-07 18458057 PMC2447139

[125] Lionaki E de Marcos Lousa C Baud C Vougioukalaki M Panayotou G Tokatlidis K. The essential function of Tim12 in vivo is ensured by the assembly interactions of its C-terminal domain. J Biol Chem. (2008) 283:15747–53. 10.1074/jbc.M800350200 18387953 PMC3259664

[126] Gebert N Gebert M Oeljeklaus S von der Malsburg K Stroud DA Kulawiak B. Dual function of Sdh3 in the respiratory chain and TIM22 protein translocase of the mitochondrial inner membrane. Mol Cell. (2011) 44:811–8. 10.1016/j.molcel.2011.09.025 22152483

[127] Kulawiak B Hopker J Gebert M Guiard B Wiedemann N Gebert N. The mitochondrial protein import machinery has multiple connections to the respiratory chain. Biochim Biophys Acta. (2013) 1827:612–26. 10.1016/j.bbabio.2012.12.004 23274250

[128] Durigon R Wang Q Ceh Pavia E Grant CM Lu H. Cytosolic thioredoxin system facilitates the import of mitochondrial small Tim proteins. EMBO Rep. (2012) 13:916–22. 10.1038/embor.2012.116 22878414 PMC3463972

[129] Allen S Balabanidou V Sideris DP Lisowsky T Tokatlidis K. Erv1 mediates the Mia40-dependent protein import pathway and provides a functional link to the respiratory chain by shuttling electrons to cytochrome c. J Mol Biol. (2005) 353:937–44. 10.1016/j.jmb.2005.08.049 16185707

[130] Mesecke N Terziyska N Kozany C Baumann F Neupert W Hell K. disulfide relay system in the intermembrane space of mitochondria that mediates protein import. Cell. (2005) 121:1059–69. 10.1016/j.cell.2005.04.011 15989955

[131] Hell K. The Erv1-Mia40 disulfide relay system in the intermembrane space of mitochondria. Biochim Biophys Acta. (2008) 1783:601–9. 10.1016/j.bbamcr.2007.12.005 18179776

[132] Rissler M Wiedemann N Pfannschmidt S Gabriel K Guiard B Pfanner N. The essential mitochondrial protein Erv1 cooperates with Mia40 in biogenesis of intermembrane space proteins. J Mol Biol. (2005) 353:485–92. 10.1016/j.jmb.2005.08.051 16181637

[133] Chacinska A Pfannschmidt S Wiedemann N Kozjak V Sanjuan Szklarz LK Schulze-Specking A. Essential role of Mia40 in import and assembly of mitochondrial intermembrane space proteins. EMBO J. (2004) 23:3735–46. 10.1038/sj.emboj.7600389 15359280 PMC522791

[134] Terziyska N Grumbt B Kozany C Hell K. Structural and functional roles of the conserved cysteine residues of the redox-regulated import receptor Mia40 in the intermembrane space of mitochondria. J Biol Chem. (2009) 284:1353–63. 10.1074/jbc.M805035200 19011240

[135] Banci L Bertini I Cefaro C Cenacchi L Ciofi-Baffoni S Felli IC. Molecular chaperone function of Mia40 triggers consecutive induced folding steps of the substrate in mitochondrial protein import. Proc Natl Acad Sci USA. (2010) 107:20190–5. 10.1073/pnas.1010095107 21059946 PMC2996643

[136] von der Malsburg K Muller JM Bohnert M Oeljeklaus S Kwiatkowska P Becker T. Dual role of mitofilin in mitochondrial membrane organization and protein biogenesis. Dev Cell. (2011) 21:694–707. 10.1016/j.devcel.2011.08.026 21944719

[137] Weckbecker D Longen S Riemer J Herrmann JM. Atp23 biogenesis reveals a chaperone-like folding activity of Mia40 in the IMS of mitochondria. EMBO J. (2012) 31:4348–58. 10.1038/emboj.2012.263 22990235 PMC3501227

[138] Peleh V Cordat E Herrmann JM. Mia40 is a trans-site receptor that drives protein import into the mitochondrial intermembrane space by hydrophobic substrate binding. Elife. (2016) 5:16177. 10.7554/eLife.16177 27343349 PMC4951193

[139] Milenkovic D Ramming T Muller JM Wenz LS Gebert N Schulze-Specking A. Identification of the signal directing Tim9 and Tim10 into the intermembrane space of mitochondria. Mol Biol Cell. (2009) 20:2530–9. 10.1091/mbc.e08-11-1108 19297525 PMC2682594

[140] Sideris DP Petrakis N Katrakili N Mikropoulou D Gallo A Ciofi-Baffoni S. A novel intermembrane space-targeting signal docks cysteines onto Mia40 during mitochondrial oxidative folding. J Cell Biol. (2009) 187:1007–22. 10.1083/jcb.200905134 20026652 PMC2806287

[141] Bien M Longen S Wagener N Chwalla I Herrmann JM Riemer J. Mitochondrial disulfide bond formation is driven by intersubunit electron transfer in Erv1 and proofread by glutathione. Mol Cell. (2010) 37:516–28. 10.1016/j.molcel.2010.01.017 20188670

[142] Bihlmaier K Mesecke N Terziyska N Bien M Hell K Herrmann JM. The disulfide relay system of mitochondria is connected to the respiratory chain. J Cell Biol. (2007) 179:389–95. 10.1083/jcb.200707123 17967948 PMC2064786

[143] Dabir DV Leverich EP Kim SK Tsai FD Hirasawa M Knaff DB. role for cytochrome c and cytochrome c peroxidase in electron shuttling from Erv1. EMBO J. (2007) 26:4801–11. 10.1038/sj.emboj.7601909 17972915 PMC2099471

[144] Kawano S Yamano K Naoe M Momose T Terao K Nishikawa S. Structural basis of yeast Tim40/Mia40 as an oxidative translocator in the mitochondrial intermembrane space. Proc Natl Acad Sci USA. (2009) 106:14403–7. 10.1073/pnas.0901793106 19667201 PMC2732879

[145] Longen S Woellhaf MW Petrungaro C Riemer J Herrmann JM. The disulfide relay of the intermembrane space oxidizes the ribosomal subunit mrp10 on its transit into the mitochondrial matrix. Dev Cell. (2014) 28:30–42. 10.1016/j.devcel.2013.11.007 24360785

[146] Jores T Klinger A Gross LE Kawano S Flinner N Duchardt-Ferner E. Characterization of the targeting signal in mitochondrial beta-barrel proteins. Nat Commun. (2016) 7:12036. 10.1038/ncomms12036 27345737 PMC4931251

[147] Klein A Israel L Lackey SW Nargang FE Imhof A Baumeister W. Characterization of the insertase for beta-barrel proteins of the outer mitochondrial membrane. J Cell Biol. (2012) 199:599–611. 10.1083/jcb.201207161 23128244 PMC3494861

[148] Wiedemann N Kozjak V Chacinska A Schonfisch B Rospert S Ryan MT. Machinery for protein sorting and assembly in the mitochondrial outer membrane. Nature. (2003) 424:565–71. 10.1038/nature01753 12891361

[149] Paschen SA Waizenegger T Stan T Preuss M Cyrklaff M Hell K. Evolutionary conservation of biogenesis of beta-barrel membrane proteins. Nature. (2003) 426:862–6. 10.1038/nature02208 14685243

[150] Hohr AIC Lindau C Wirth C Qiu J Stroud DA Kutik S. Membrane protein insertion through a mitochondrial beta-barrel gate. Science. (2018) 359:aah6834. 10.1126/science.aah6834 29348211 PMC5959003

[151] Kutik S Stojanovski D Becker L Becker T Meinecke M Kruger V. Dissecting membrane insertion of mitochondrial beta-barrel proteins. Cell. (2008) 132:1011–24. 10.1016/j.cell.2008.01.028 18358813

[152] Wenz LS Ellenrieder L Qiu J Bohnert M Zufall N van der Laan M. Sam37 is crucial for formation of the mitochondrial TOM-SAM supercomplex, thereby promoting beta-barrel biogenesis. J Cell Biol. (2015) 210:1047–54. 10.1083/jcb.201504119 26416958 PMC4586741

[153] Popov-Celeketic J Waizenegger T Rapaport D. Mim1 functions in an oligomeric form to facilitate the integration of Tom20 into the mitochondrial outer membrane. J Mol Biol. (2008) 376:671–80. 10.1016/j.jmb.2007.12.006 18177669

[154] Hulett JM Lueder F Chan NC Perry AJ Wolynec P Likic VA. The transmembrane segment of Tom20 is recognized by Mim1 for docking to the mitochondrial TOM complex. J Mol Biol. (2008) 376:694–704. 10.1016/j.jmb.2007.12.021 18187149

[155] Papic D Krumpe K Dukanovic J Dimmer KS Rapaport D. Multispan mitochondrial outer membrane protein Ugo1 follows a unique Mim1-dependent import pathway. J Cell Biol. (2011) 194:397–405. 10.1083/jcb.201102041 21825074 PMC3153653

[156] Becker T Wenz LS Kruger V Lehmann W Muller JM Goroncy L. The mitochondrial import protein Mim1 promotes biogenesis of multispanning outer membrane proteins. J Cell Biol. (2011) 194:387–95. 10.1083/jcb.201102044 21825073 PMC3153637

[157] Kruger V Becker T Becker L Montilla-Martinez M Ellenrieder L Vogtle FN. Identification of new channels by systematic analysis of the mitochondrial outer membrane. J Cell Biol. (2017) 216:3485–95. 10.1083/jcb.201706043 28916712 PMC5674900

[158] Dimmer KS Papic D Schumann B Sperl D Krumpe K Walther DM. A crucial role for Mim2 in the biogenesis of mitochondrial outer membrane proteins. J Cell Sci. (2012) 125:3464–73. 10.1242/jcs.103804 22467864

[159] Gupta A Becker T. Mechanisms and pathways of mitochondrial outer membrane protein biogenesis. Biochim Biophys Acta Bioenerg. (2021) 1862:148323. 10.1016/j.bbabio.2020.148323 33035511

[160] Keskin A Akdogan E Dunn CD. Evidence for amino acid snorkeling from a high-resolution, *in vivo* analysis of Fis1 tail-anchor insertion at the mitochondrial outer membrane. Genetics. (2017) 205:691–705. 10.1534/genetics.116.196428 28007883 PMC5289845

[161] Vogtle FN Keller M Taskin AA Horvath SE Guan XL Prinz C. The fusogenic lipid phosphatidic acid promotes the biogenesis of mitochondrial outer membrane protein Ugo1. J Cell Biol. (2015) 210:951–60. 10.1083/jcb.201506085 26347140 PMC4576865

[162] Sauerwald J Jores T Eisenberg-Bord M Chuartzman SG Schuldiner M Rapaport D. Genome-wide screens in saccharomyces cerevisiae highlight a role for cardiolipin in biogenesis of mitochondrial outer membrane multispan proteins. Mol Cell Biol. (2015) 35:3200–11. 10.1128/MCB.00107-15 26149385 PMC4539369

[163] Haynes CM Ron D. The mitochondrial UPR - protecting organelle protein homeostasis. J Cell Sci. (2010) 123:3849–55. 10.1242/jcs.075119 21048161

[164] Topf U Suppanz I Samluk L Wrobel L Boser A Sakowska P. Quantitative proteomics identifies redox switches for global translation modulation by mitochondrially produced reactive oxygen species. Nat Commun. (2018) 9:324. 10.1038/s41467-017-02694-8 29358734 PMC5778013

[165] Mehnert CS Rampelt H Gebert M Oeljeklaus S Schrempp SG Kochbeck L. The mitochondrial ADP/ATP carrier associates with the inner membrane presequence translocase in a stoichiometric manner. J Biol Chem. (2014) 289:27352–62. 10.1074/jbc.M114.556498 25124039 PMC4175365

[166] Dennerlein S Oeljeklaus S Jans D Hellwig C Bareth B Jakobs S. MITRAC7 acts as a COX1-specific chaperone and reveals a checkpoint during cytochrome c oxidase assembly. Cell Rep. (2015) 12:1644–55. 10.1016/j.celrep.2015.08.009 26321642

[167] Mick DU Dennerlein S Wiese H Reinhold R Pacheu-Grau D Lorenzi I. MITRAC links mitochondrial protein translocation to respiratory-chain assembly and translational regulation. Cell. (2012) 151:1528–41. 10.1016/j.cell.2012.11.053 23260140

[168] Richter-Dennerlein R Oeljeklaus S Lorenzi I Ronsor C Bareth B Schendzielorz AB. Mitochondrial protein synthesis adapts to influx of nuclear-encoded protein. Cell. (2016) 167:471–83 e10. 10.1016/j.cell.2016.09.003 27693358 PMC5055049

[169] Shpilka T Haynes CM. The mitochondrial UPR: mechanisms, physiological functions and implications in ageing. Nat Rev Mol Cell Biol. (2018) 19:109–20. 10.1038/nrm.2017.110 29165426

[170] Nargund AM Pellegrino MW Fiorese CJ Baker BM Haynes CM. Mitochondrial import efficiency of ATFS-1 regulates mitochondrial UPR activation. Science. (2012) 337:587–90. 10.1126/science.1223560 22700657 PMC3518298

[171] Rolland SG Schneid S Schwarz M Rackles E Fischer C Haeussler S. Compromised mitochondrial protein import acts as a signal for UPR(mt). Cell Rep. (2019) 28:1659–69 e5. 10.1016/j.celrep.2019.07.049 31412237

[172] Wrobel L Topf U Bragoszewski P Wiese S Sztolsztener ME Oeljeklaus S. Mistargeted mitochondrial proteins activate a proteostatic response in the cytosol. Nature. (2015) 524:485–8. 10.1038/nature14951 26245374

[173] Wang X Chen XJ. A cytosolic network suppressing mitochondria-mediated proteostatic stress and cell death. Nature. (2015) 524:481–4. 10.1038/nature14859 26192197 PMC4582408

[174] Jin SM Lazarou M Wang C Kane LA Narendra DP Youle RJ. Mitochondrial membrane potential regulates PINK1 import and proteolytic destabilization by PARL. J Cell Biol. (2010) 191:933–42. 10.1083/jcb.201008084 21115803 PMC2995166

[175] Yamano K Youle RJ. PINK1 is degraded through the N-end rule pathway. Autophagy. (2013) 9:1758–69. 10.4161/auto.24633 24121706 PMC4028335

[176] Lazarou M Jin SM Kane LA Youle RJ. Role of PINK1 binding to the TOM complex and alternate intracellular membranes in recruitment and activation of the E3 ligase Parkin. Dev Cell. (2012) 22:320–33. 10.1016/j.devcel.2011.12.014 22280891 PMC3288275

[177] Kane LA Lazarou M Fogel AI Li Y Yamano K Sarraf SA Banerjee S. PINK1 phosphorylates ubiquitin to activate Parkin E3 ubiquitin ligase activity. J Cell Biol. (2014) 205:143–53. 10.1083/jcb.201402104 24751536 PMC4003245

[178] Ruan L Zhou C Jin E Kucharavy A Zhang Y Wen Z. Cytosolic proteostasis through importing of misfolded proteins into mitochondria. Nature. (2017) 543:443–46. 10.1038/nature21695 28241148 PMC5793917

[179] Wagner I Arlt H van Dyck L Langer T Neupert W. Molecular chaperones cooperate with PIM1 protease in the degradation of misfolded proteins in mitochondria. EMBO J. (1994) 13:5135–45. 10.1002/j.1460-2075.1994.tb06843.x 7957078 PMC395461

[180] Kang SG Dimitrova MN Ortega J Ginsburg A Maurizi MR. Human mitochondrial ClpP is a stable heptamer that assembles into a tetradecamer in the presence of ClpX. J Biol Chem. (2005) 280:35424–32. 10.1074/jbc.M507240200 16115876

[181] Bender T Lewrenz I Franken S Baitzel C Voos W. Mitochondrial enzymes are protected from stress-induced aggregation by mitochondrial chaperones and the Pim1/LON protease. Mol Biol Cell. (2011) 22:541–54. 10.1091/mbc.e10-08-0718 21209324 PMC3046053

[182] Fischer F Langer JD Osiewacz HD. Identification of potential mitochondrial CLPXP protease interactors and substrates suggests its central role in energy metabolism. Sci Rep. (2015) 5:18375. 10.1038/srep18375 26679294 PMC4683621

[183] Ohba Y MacVicar T Langer T. Regulation of mitochondrial plasticity by the i-AAA protease YME1L. Biol Chem. (2020) 401:877–90. 10.1515/hsz-2020-0120 32087062

[184] Baker MJ Mooga VP Guiard B Langer T Ryan MT Stojanovski D. Impaired folding of the mitochondrial small TIM chaperones induces clearance by the i-AAA protease. J Mol Biol. (2012) 424:227–39. 10.1016/j.jmb.2012.09.019 23036860

[185] Wu X Li L Jiang H. Mitochondrial inner-membrane protease Yme1 degrades outer-membrane proteins Tom22 and Om45. J Cell Biol. (2018) 217:139–49. 10.1083/jcb.201702125 29138251 PMC5748973

[186] Deshwal S Fiedler KU Langer T. Mitochondrial proteases: multifaceted regulators of mitochondrial plasticity. Annu Rev Biochem. (2020) 89:501–28. 10.1146/annurev-biochem-062917-012739 32075415

[187] Opalinska M Janska H. AAA proteases: guardians of mitochondrial function and homeostasis. Cells. (2018) 7:100163. 10.3390/cells7100163 30314276 PMC6210556

[188] Hoppins S Collins SR Cassidy-Stone A Hummel E Devay RM Lackner LL. A mitochondrial-focused genetic interaction map reveals a scaffold-like complex required for inner membrane organization in mitochondria. J Cell Biol. (2011) 195:323–40. 10.1083/jcb.201107053 21987634 PMC3198156

[189] Harner M Korner C Walther D Mokranjac D Kaesmacher J Welsch U. The mitochondrial contact site complex, a determinant of mitochondrial architecture. EMBO J. (2011) 30:4356–70. 10.1038/emboj.2011.379 22009199 PMC3230385

[190] Pfanner N van der Laan M Amati P Capaldi RA Caudy AA Chacinska A. Uniform nomenclature for the mitochondrial contact site and cristae organizing system. J Cell Biol. (2014) 204:1083–6. 10.1083/jcb.201401006 24687277 PMC3971754

[191] Bohnert M Wenz LS Zerbes RM Horvath SE Stroud DA von der Malsburg K. Role of mitochondrial inner membrane organizing system in protein biogenesis of the mitochondrial outer membrane. Mol Biol Cell. (2012) 23:3948–56. 10.1091/mbc.e12-04-0295 22918945 PMC3469511

[192] Ehses S Raschke I Mancuso G Bernacchia A Geimer S Tondera D. Regulation of OPA1 processing and mitochondrial fusion by m-AAA protease isoenzymes and OMA1. J Cell Biol. (2009) 187:1023–36. 10.1083/jcb.200906084 20038678 PMC2806285

[193] Ishihara N Fujita Y Oka T Mihara K. Regulation of mitochondrial morphology through proteolytic cleavage of OPA1. EMBO J. (2006) 25:2966–77. 10.1038/sj.emboj.7601184 16778770 PMC1500981

[194] Anand R Wai T Baker MJ Kladt N Schauss AC Rugarli E. The i-AAA protease YME1L and OMA1 cleave OPA1 to balance mitochondrial fusion and fission. J Cell Biol. (2014) 204:919–29. 10.1083/jcb.201308006 24616225 PMC3998800

[195] Wai T Garcia-Prieto J Baker MJ Merkwirth C Benit P Rustin P. Imbalanced OPA1 processing and mitochondrial fragmentation cause heart failure in mice. Science. (2015) 350:aad0116. 10.1126/science.aad0116 26785494

[196] Ellenrieder L Opalinski L Becker L Kruger V Mirus O Straub SP. Separating mitochondrial protein assembly and endoplasmic reticulum tethering by selective coupling of Mdm10. Nat Commun. (2016) 7:13021. 10.1038/ncomms13021 27721450 PMC5476798

[197] Stroud DA Oeljeklaus S Wiese S Bohnert M Lewandrowski U Sickmann A. Composition and topology of the endoplasmic reticulum-mitochondria encounter structure. J Mol Biol. (2011) 413:743–50. 10.1016/j.jmb.2011.09.012 21945531

[198] Muller CS Bildl W Haupt A Ellenrieder L Becker T Hunte C. Cryo-slicing blue native-mass spectrometry (csBN-MS), a novel technology for high resolution complexome profiling. Mol Cell Proteomics. (2016) 15:669–81. 10.1074/mcp.M115.054080 26598645 PMC4739680

[199] Elbaz-Alon Y Eisenberg-Bord M Shinder V Stiller SB Shimoni E Wiedemann N. Lam6 regulates the extent of contacts between organelles. Cell Rep. (2015) 12:7–14. 10.1016/j.celrep.2015.06.022 26119743 PMC4518459

[200] Murley A Sarsam RD Toulmay A Yamada J Prinz WA Nunnari J. Ltc1 is an ER-localized sterol transporter and a component of ER-mitochondria and ER-vacuole contacts. J Cell Biol. (2015) 209:539–48. 10.1083/jcb.201502033 25987606 PMC4442815

[201] Meisinger C Wiedemann N Rissler M Strub A Milenkovic D Schonfisch B. Mitochondrial protein sorting: differentiation of beta-barrel assembly by Tom7-mediated segregation of Mdm10. J Biol Chem. (2006) 281:22819–26. 10.1074/jbc.M602679200 16760475

[202] Kornmann B Currie E Collins SR Schuldiner M Nunnari J Weissman JS. An ER-mitochondria tethering complex revealed by a synthetic biology screen. Science. (2009) 325:477–81. 10.1126/science.1175088 19556461 PMC2933203

[203] Meisinger C Rissler M Chacinska A Szklarz LK Milenkovic D Kozjak V. The mitochondrial morphology protein Mdm10 functions in assembly of the preprotein translocase of the outer membrane. Dev Cell. (2004) 7:61–71. 10.1016/j.devcel.2004.06.003 15239954

[204] Yamano K Tanaka-Yamano S Endo T. Mdm10 as a dynamic constituent of the TOB/SAM complex directs coordinated assembly of Tom40. EMBO Rep. (2010) 11:187–93. 10.1038/embor.2009.283 20111053 PMC2838686

[205] Flinner N Ellenrieder L Stiller SB Becker T Schleiff E Mirus O. Mdm10 is an ancient eukaryotic porin co-occurring with the ERMES complex. Biochim Biophys Acta. (2013) 1833:3314–25. 10.1016/j.bbamcr.2013.10.006 24135058

[206] Boengler K Gres P Cabestrero A Ruiz-Meana M Garcia-Dorado D Heusch G. Prevention of the ischemia-induced decrease in mitochondrial Tom20 content by ischemic preconditioning. J Mol Cell Cardiol. (2006) 41:426–30. 10.1016/j.yjmcc.2006.05.015 16828795

[207] Feng R Cai M Wang X Zhang J Tian Z. Early aerobic exercise combined with hydrogen-rich saline as preconditioning protects myocardial injury induced by acute myocardial infarction in rats. Appl Biochem Biotechnol. (2019) 187:663–76. 10.1007/s12010-018-2841-0 30033489

[208] Budas GR Churchill EN Disatnik MH Sun L Mochly-Rosen D. Mitochondrial import of PKCepsilon is mediated by HSP90: a role in cardioprotection from ischaemia and reperfusion injury. Cardiovasc Res. (2010) 88:83–92. 10.1093/cvr/cvq154 20558438 PMC2936125

[209] Srisakuldee W Makazan Z Nickel BE Zhang F Thliveris JA Pasumarthi KB. The FGF-2-triggered protection of cardiac subsarcolemmal mitochondria from calcium overload is mitochondrial connexin 43-dependent. Cardiovasc Res. (2014) 103:72–80. 10.1093/cvr/cvu066 24654232

[210] Paillard M Tubbs E Thiebaut PA Gomez L Fauconnier J Da Silva CC. Depressing mitochondria-reticulum interactions protects cardiomyocytes from lethal hypoxia-reoxygenation injury. Circulation. (2013) 128:1555–65. 10.1161/CIRCULATIONAHA.113.001225 23983249

[211] Zhang J Li M Zhang Z Zhu R Olcese R Stefani E. The mitochondrial BKCa channel cardiac interactome reveals BKCa association with the mitochondrial import receptor subunit Tom22, and the adenine nucleotide translocator. Mitochondrion. (2017) 33:84–101. 10.1016/j.mito.2016.08.017 27592226 PMC5332438

[212] Bose HS Whittal RM Marshall B Rajapaksha M Wang NP Bose M. A novel mitochondrial complex of aldosterone synthase, steroidogenic acute regulatory protein, and Tom22 synthesizes aldosterone in the rat heart. J Pharmacol Exp Ther. (2021) 377:108–20. 10.1124/jpet.120.000365 33526603

[213] Benak D Sotakova-Kasparova D Neckar J Kolar F Hlavackova M. Selection of optimal reference genes for gene expression studies in chronically hypoxic rat heart. Mol Cell Biochem. (2019) 461:15–22. 10.1007/s11010-019-03584-x 31300984

[214] Middelberg RP Ferreira MA Henders AK Heath AC Madden PA Montgomery GW. Genetic variants in LPL, OASL and TOMM40/APOE-C1-C2-C4 genes are associated with multiple cardiovascular-related traits. BMC Med Genet. (2011) 12:123. 10.1186/1471-2350-12-123 21943158 PMC3189113

[215] Jeemon P Pettigrew K Sainsbury C Prabhakaran D Padmanabhan S. Implications of discoveries from genome-wide association studies in current cardiovascular practice. World J Cardiol. (2011) 3:230–47. 10.4330/wjc.v3.i7.230 21860704 PMC3158871

[216] Yang Z Sun W Hu K. Molecular mechanism underlying adenosine receptor-mediated mitochondrial targeting of protein kinase C. Biochim Biophys Acta. (2012) 1823:950–8. 10.1016/j.bbamcr.2011.12.012 22233927

[217] Kang C Qin J Osei W Hu K. Regulation of protein kinase C-epsilon and its age-dependence. Biochem Biophys Res Commun. (2017) 482:1201–06. 10.1016/j.bbrc.2016.12.012 27919679

[218] Li J Qi M Li C Shi D Zhang D Xie D. Tom70 serves as a molecular switch to determine pathological cardiac hypertrophy. Cell Res. (2014) 24:977–93. 10.1038/cr.2014.94 25022898 PMC4123302

[219] Pei HF Hou JN Wei FP Xue Q Zhang F Peng CF. Melatonin attenuates postmyocardial infarction injury *via* increasing Tom70 expression. J Pineal Res. (2017) 62:12371. 10.1111/jpi.12371 27706848

[220] Lochner A Marais E Huisamen B. Melatonin and cardioprotection against ischaemia/reperfusion injury: what's new? A review. J Pineal Res. (2018) 65:e12490. 10.1111/jpi.12490 29570845

[221] Kato H Lu Q Rapaport D Kozjak-Pavlovic V. Tom70 is essential for PINK1 import into mitochondria. PLoS ONE. (2013) 8:e58435. 10.1371/journal.pone.0058435 23472196 PMC3589387

[222] Xue Q Pei H Liu Q Zhao M Sun J Gao E. MICU1 protects against myocardial ischemia/reperfusion injury and its control by the importer receptor Tom70. Cell Death Dis. (2017) 8:e2923. 10.1038/cddis.2017.280 28703803 PMC5550843

[223] Garg V Hu K. Protein kinase C isoform-dependent modulation of ATP-sensitive K+ channels in mitochondrial inner membrane. Am J Physiol Heart Circ Physiol. (2007) 293:H322–32. 10.1152/ajpheart.01035.2006 17351068

[224] Mayr M Liem D Zhang J Li X Avliyakulov NK Yang JI. Proteomic and metabolomic analysis of cardioprotection: Interplay between protein kinase C epsilon and delta in regulating glucose metabolism of murine hearts. J Mol Cell Cardiol. (2009) 46:268–77. 10.1016/j.yjmcc.2008.10.008 19027023 PMC3661410

[225] Yuan Y Pan SS. Parkin mediates mitophagy to participate in cardioprotection induced by late exercise preconditioning but Bnip3 does not. J Cardiovasc Pharmacol. (2018) 71:303–16. 10.1097/FJC.0000000000000572 29538088

[226] Wang P Wang D Yang Y Hou J Wan J Ran F. Tom70 protects against diabetic cardiomyopathy through its antioxidant and antiapoptotic properties. Hypertens Res. (2020) 43:1047–56. 10.1038/s41440-020-0518-x 32724135

[227] Botta A Laher I Beam J Decoffe D Brown K Halder S. Short term exercise induces PGC-1alpha, ameliorates inflammation and increases mitochondrial membrane proteins but fails to increase respiratory enzymes in aging diabetic hearts. PLoS ONE. (2013) 8:e70248. 10.1371/journal.pone.0070248 23936397 PMC3731348

[228] Giorgianni F Usman Khan M Weber KT Gerling IC Beranova-Giorgianni S. Phosphoproteome mapping of cardiomyocyte mitochondria in a rat model of heart failure. Mol Cell Biochem. (2014) 389:159–67. 10.1007/s11010-013-1937-7 24395194 PMC4009925

[229] Palmer ND Kahali B Kuppa A Chen Y Du X Feitosa MF. Allele specific variation at APOE increases non-alcoholic fatty liver disease and obesity but decreases risk of Alzheimer's disease and myocardial infarction. Hum Mol Genet. (2021) 2021:ddab096. 10.1093/hmg/ddab096 33856023 PMC8283205

[230] Talmud PJ Drenos F Shah S Shah T Palmen J Verzilli C. Gene-centric association signals for lipids and apolipoproteins identified via the HumanCVD BeadChip. Am J Hum Genet. (2009) 85:628–42. 10.1016/j.ajhg.2009.10.014 19913121 PMC2775832

[231] Kong X Zhao Q Xing X Zhang B Zhang X Hong J. Genetic variants associated with lipid profiles in Chinese patients with type 2 diabetes. PLoS ONE. (2015) 10:e0135145. 10.1371/journal.pone.0135145 26252223 PMC4529182

[232] Abe S Tokoro F Matsuoka R Arai M Noda T Watanabe S. Association of genetic variants with dyslipidemia. Mol Med Rep. (2015) 12:5429–36. 10.3892/mmr.2015.4081 26238946

[233] Park S Kang S. A minor allele of the haplotype located in the 19q13 loci is associated with a decreased risk of hyper-LDL-cholesterolemia, and a balanced diet and high protein intake can reduce the risk. Lipids Health Dis. (2020) 19:178. 10.1186/s12944-020-01352-1 32727492 PMC7391697

[234] Christiansen MK Larsen SB Nyegaard M Neergaard-Petersen S Ajjan R Wurtz M. Coronary artery disease-associated genetic variants and biomarkers of inflammation. PLoS ONE. (2017) 12:e0180365. 10.1371/journal.pone.0180365 28686695 PMC5501546

[235] Kraja AT Chasman DI North KE Reiner AP Yanek LR Kilpelainen TO. Pleiotropic genes for metabolic syndrome and inflammation. Mol Genet Metab. (2014) 112:317–38. 10.1016/j.ymgme.2014.04.007 24981077 PMC4122618

[236] Zeh R. Neurological and Molecular Biological Characterisation of the Mutant Mouse Line Tom40. Munich: Technische Universität München. (2013).

[237] Wang X Wang S Liu W Wang T Wang J Gao X. Epigenetic upregulation of miR-126 induced by heat stress contributes to apoptosis of rat cardiomyocytes by promoting Tomm40 transcription. J Mol Cell Cardiol. (2019) 129:39–48. 10.1016/j.yjmcc.2018.10.005 30296408

[238] Barcena ML Pozdniakova S Haritonow N Breiter P Kuhl AA Milting H. Dilated cardiomyopathy impairs mitochondrial biogenesis and promotes inflammation in an age- and sex-dependent manner. Aging. (2020) 12:24117–33. 10.18632/aging.202283 33303703 PMC7762497

[239] Yeo A Li L Warren L Aponte J Fraser D King K. Pharmacogenetic meta-analysis of baseline risk factors, pharmacodynamic, efficacy and tolerability endpoints from two large global cardiovascular outcomes trials for darapladib. PLoS ONE. (2017) 12:e0182115. 10.1371/journal.pone.0182115 28753643 PMC5533343

[240] Guo Y Cheong N Zhang Z De Rose R Deng Y Farber SA. Tim50, a component of the mitochondrial translocator, regulates mitochondrial integrity and cell death. J Biol Chem. (2004) 279:24813–25. 10.1074/jbc.M402049200 15044455

[241] Tang K Zhao Y Li H Zhu M Li W Liu W. Translocase of inner membrane 50 functions as a novel protective regulator of pathological cardiac hypertrophy. J Am Heart Assoc. (2017) 6:4346. 10.1161/JAHA.116.004346 28432072 PMC5532988

[242] Zhang XX Wu XS Mi SH Fang SJ Liu S Xin Y. Neuregulin-1 promotes mitochondrial biogenesis, attenuates mitochondrial dysfunction, and prevents hypoxia/reoxygenation injury in neonatal cardiomyocytes. Cell Biochem Funct. (2020) 38:549–57. 10.1002/cbf.3503 32037595

[243] Schubert C Raparelli V Westphal C Dworatzek E Petrov G Kararigas G. Reduction of apoptosis and preservation of mitochondrial integrity under ischemia/reperfusion injury is mediated by estrogen receptor beta. Biol Sex Differ. (2016) 7:53. 10.1186/s13293-016-0104-8 27688871 PMC5035458

[244] Bian X Teng T Zhao H Qin J Qiao Z Sun Y. Zinc prevents mitochondrial superoxide generation by inducing mitophagy in the setting of hypoxia/reoxygenation in cardiac cells. Free Radic Res. (2018) 52:80–91. 10.1080/10715762.2017.1414949 29216769

[245] Williamson CL Dabkowski ER Dillmann WH Hollander JM. Mitochondria protection from hypoxia/reoxygenation injury with mitochondria heat shock protein 70 overexpression. Am J Physiol Heart Circ Physiol. (2008) 294:H249–56. 10.1152/ajpheart.00775.2007 17982016

[246] Zhou TT Wang XY Huang J Deng YZ Qiu LJ Liu HY. Mitochondrial translocation of DJ-1 is mediated by Grp75: implication in cardioprotection of resveratrol against hypoxia/reoxygenation-induced oxidative stress. J Cardiovasc Pharmacol. (2020) 75:305–13. 10.1097/FJC.0000000000000805 32040033

[247] Baseler WA Dabkowski ER Williamson CL Croston TL Thapa D Powell MJ. Proteomic alterations of distinct mitochondrial subpopulations in the type 1 diabetic heart: contribution of protein import dysfunction. Am J Physiol Regul Integr Comp Physiol. (2011) 300:R186–200. 10.1152/ajpregu.00423.2010 21048079 PMC3043804

[248] Shepherd DL Hathaway QA Nichols CE Durr AJ Pinti MV Hughes KM. Mitochondrial proteome disruption in the diabetic heart through targeted epigenetic regulation at the mitochondrial heat shock protein 70 (mtHsp70) nuclear locus. J Mol Cell Cardiol. (2018) 119:104–15. 10.1016/j.yjmcc.2018.04.016 29733819 PMC5987221

[249] Kirmanoglou K Hannekum A Schafler AE. Expression of mortalin in patients with chronic atrial fibrillation. Basic Res Cardiol. (2004) 99:404–8. 10.1007/s00395-004-0477-4 15309412

[250] Davey KM Parboosingh JS McLeod DR Chan A Casey R Ferreira P. Mutation of DNAJC19, a human homologue of yeast inner mitochondrial membrane co-chaperones, causes DCMA syndrome, a novel autosomal recessive Barth syndrome-like condition. J Med Genet. (2006) 43:385–93. 10.1136/jmg.2005.036657 16055927 PMC2564511

[251] Ojala T Polinati P Manninen T Hiippala A Rajantie J Karikoski R. New mutation of mitochondrial DNAJC19 causing dilated and noncompaction cardiomyopathy, anemia, ataxia, and male genital anomalies. Pediatr Res. (2012) 72:432–7. 10.1038/pr.2012.92 22797137

[252] Ucar SK Mayr JA Feichtinger RG Canda E Coker M Wortmann SB. Previously unreported biallelic mutation in DNAJC19: are sensorineural hearing loss and basal ganglia lesions additional features of dilated cardiomyopathy and ataxia (DCMA) syndrome? JIMD Rep. (2017) 35:39–45. 10.1007/8904_2016_23 27928778 PMC5585102

[253] Al Teneiji A Siriwardena K George K Mital S Mercimek-Mahmutoglu S. Progressive cerebellar atrophy and a novel homozygous pathogenic DNAJC19 variant as a cause of dilated cardiomyopathy ataxia syndrome. Pediatr Neurol. (2016) 62:58–61. 10.1016/j.pediatrneurol.2016.03.020 27426421

[254] Sparkes R Patton D Bernier F. Cardiac features of a novel autosomal recessive dilated cardiomyopathic syndrome due to defective importation of mitochondrial protein. Cardiol Young. (2007) 17:215–7. 10.1017/S1047951107000042 17244376

[255] Vasilescu C Ojala TH Brilhante V Ojanen S Hinterding HM Palin E. Genetic basis of severe childhood-onset cardiomyopathies. J Am Coll Cardiol. (2018) 72:2324–38. 10.1016/j.jacc.2018.08.2171 30384889

[256] Mehawej C Delahodde A Legeai-Mallet L Delague V Kaci N Desvignes JP. The impairment of MAGMAS function in human is responsible for a severe skeletal dysplasia. PLoS Genet. (2014) 10:e1004311. 10.1371/journal.pgen.1004311 24786642 PMC4006740

[257] Rodriguez-Sinovas A Boengler K Cabestrero A Gres P Morente M Ruiz-Meana M. Translocation of connexin 43 to the inner mitochondrial membrane of cardiomyocytes through the heat shock protein 90-dependent TOM pathway and its importance for cardioprotection. Circ Res. (2006) 99:93–101. 10.1161/01.RES.0000230315.56904.de 16741159

[258] Nguyen T Wong R Wang G Gucek M Steenbergen C Murphy E. Acute inhibition of GSK causes mitochondrial remodeling. Am J Physiol Heart Circ Physiol. (2012) 302:H2439–45. 10.1152/ajpheart.00033.2012 22467305 PMC3378293

[259] Small BA Lu Y Hsu AK Gross GJ Gross ER. Morphine reduces myocardial infarct size *via* heat shock protein 90 in rodents. Biomed Res Int. (2015) 2015:129612. 10.1155/2015/129612 26413502 PMC4564588

[260] Tu RH Li QJ Huang Z He Y Meng JJ Zheng HL Zeng ZY. Novel functional role of heat shock protein 90 in mitochondrial connexin 43-mediated hypoxic postconditioning. Cell Physiol Biochem. (2017) 44:982–97. 10.1159/000485399 29179175

[261] Barksdale KA Bijur GN. The basal flux of Akt in the mitochondria is mediated by heat shock protein 90. J Neurochem. (2009) 108:1289–99. 10.1111/j.1471-4159.2009.05878.x 19187436 PMC2696161

[262] Borillo GA Mason M Quijada P Volkers M Cottage C McGregor M. Pim-1 kinase protects mitochondrial integrity in cardiomyocytes. Circ Res. (2010) 106:1265–74. 10.1161/CIRCRESAHA.109.212035 20203306 PMC2864233

[263] Fan F Duan Y Yang F Trexler C Wang H Huang L. Deletion of heat shock protein 60 in adult mouse cardiomyocytes perturbs mitochondrial protein homeostasis and causes heart failure. Cell Death Differ. (2020) 27:587–600. 10.1038/s41418-019-0374-x 31209364 PMC7205885

[264] Lin KM Hollander JM Kao VY Lin B Macpherson L Dillmann WH. Myocyte protection by 10 kD heat shock protein (Hsp10) involves the mobile loop and attenuation of the Ras GTP-ase pathway. FASEB J. (2004) 18:1004–6. 10.1096/fj.03-0348fje 15059967

[265] Schafler AE Kirmanoglou K Pecher P Hannekum A Schumacher B. Overexpression of heat shock protein 60/10 in myocardium of patients with chronic atrial fibrillation. Ann Thorac Surg. (2002) 74:767–70. 10.1016/S0003-4975(02)03830-4 12238837

[266] Agsteribbe E Huckriede A Veenhuis M Ruiters MH Niezen-Koning KE Skjeldal OH. A fatal, systemic mitochondrial disease with decreased mitochondrial enzyme activities, abnormal ultrastructure of the mitochondria and deficiency of heat shock protein 60. Biochem Biophys Res Commun. (1993) 193:146–54. 10.1006/bbrc.1993.1602 8503901

[267] Joshi M Anselm I Shi J Bale TA Towne M Schmitz-Abe K. Mutations in the substrate binding glycine-rich loop of the mitochondrial processing peptidase-alpha protein (PMPCA) cause a severe mitochondrial disease. Cold Spring Harb Mol Case Stud. (2016) 2:a000786. 10.1101/mcs.a000786 27148589 PMC4853520

[268] Eldomery MK Akdemir ZC Vogtle FN Charng WL Mulica P Rosenfeld JA. MIPEP recessive variants cause a syndrome of left ventricular non-compaction, hypotonia, and infantile death. Genome Med. (2016) 8:106. 10.1186/s13073-016-0360-6 27799064 PMC5088683

[269] Crewe C Schafer C Lee I Kinter M Szweda LI. Regulation of pyruvate dehydrogenase kinase 4 in the heart through degradation by the lon protease in response to mitochondrial substrate availability. J Biol Chem. (2017) 292:305–12. 10.1074/jbc.M116.754127 27856638 PMC5217688

[270] Hoshino A Okawa Y Ariyoshi M Kaimoto S Uchihashi M Fukai K. Oxidative post-translational modifications develop LONP1 dysfunction in pressure overload heart failure. Circ Heart Fail. (2014) 7:500–9. 10.1161/CIRCHEARTFAILURE.113.001062 24740269

[271] Zhan R Guo W Gao X Liu X Xu K Tang B. Real-time *in situ* monitoring of Lon and Caspase-3 for assessing the state of cardiomyocytes under hypoxic conditions *via* a novel Au-Se fluorescent nanoprobe. Biosens Bioelectron. (2021) 176:112965. 10.1016/j.bios.2021.112965 33421759

[272] Kuo CY Chiu YC Lee AY Hwang TL. Mitochondrial Lon protease controls ROS-dependent apoptosis in cardiomyocyte under hypoxia. Mitochondrion. (2015) 23:7–16. 10.1016/j.mito.2015.04.004 25922169

[273] Sepuri NBV Angireddy R Srinivasan S Guha M Spear J Lu B. Mitochondrial LON protease-dependent degradation of cytochrome c oxidase subunits under hypoxia and myocardial ischemia. Biochim Biophys Acta Bioenerg. (2017) 1858:519–28. 10.1016/j.bbabio.2017.04.003 28442264 PMC5507603

[274] Pomatto LCD Cline M Woodward N Pakbin P Sioutas C Morgan TE. Aging attenuates redox adaptive homeostasis and proteostasis in female mice exposed to traffic-derived nanoparticles (“vehicular smog”). Free Radic Biol Med. (2018) 121:86–97. 10.1016/j.freeradbiomed.2018.04.574 29709705 PMC5987225

[275] Delaval E Perichon M Friguet B. Age-related impairment of mitochondrial matrix aconitase and ATP-stimulated protease in rat liver and heart. Eur J Biochem. (2004) 271:4559–64. 10.1111/j.1432-1033.2004.04422.x 15560797

[276] Guillon B Bulteau AL Wattenhofer-Donze M Schmucker S Friguet B Puccio H. Frataxin deficiency causes upregulation of mitochondrial Lon and ClpP proteases and severe loss of mitochondrial Fe-S proteins. FEBS J. (2009) 276:1036–47. 10.1111/j.1742-4658.2008.06847.x 19154341

[277] Wu B Li J Ni H Zhuang X Qi Z Chen Q. TLR4 activation promotes the progression of experimental autoimmune myocarditis to dilated cardiomyopathy by inducing mitochondrial dynamic imbalance. Oxid Med Cell Longev. (2018) 2018:3181278. 10.1155/2018/3181278 30046376 PMC6038665

[278] Hu Y Xu Y Chen W Qiu Z. Stomatin-like protein-2: a potential target to treat mitochondrial cardiomyopathy. Heart Lung Circ. (2021) 5:74. 10.1016/j.hlc.2021.05.074 34088631

[279] Zhao Y Zhu J Zhang N Liu Q Wang Y Hu X. GDF11 enhances therapeutic efficacy of mesenchymal stem cells for myocardial infarction via YME1L-mediated OPA1 processing. Stem Cells Transl Med. (2020) 9:1257–71. 10.1002/sctm.20-0005 32515551 PMC7519765

[280] Seiferling D Szczepanowska K Becker C Senft K Hermans S Maiti P. Loss of CLPP alleviates mitochondrial cardiomyopathy without affecting the mammalian UPRmt. EMBO Rep. (2016) 17:953–64. 10.15252/embr.201642077 27154400 PMC4931557

[281] Mayr JA Haack TB Graf E Zimmermann FA Wieland T Haberberger B. Lack of the mitochondrial protein acylglycerol kinase causes Sengers syndrome. Am J Hum Genet. (2012) 90:314–20. 10.1016/j.ajhg.2011.12.005 22284826 PMC3276657

[282] Allali S Dorboz I Samaan S Slama A Rambaud C Boespflug-Tanguy O. Mutation in the AGK gene in two siblings with unusual Sengers syndrome. Metab Brain Dis. (2017) 32:2149–54. 10.1007/s11011-017-0101-6 28868593

[283] Khatter S Puri RD Bijarnia-Mahay S Aggarwal M Ramprasad V Saxena R. Sengers syndrome in Asian Indians–two novel mutations and variant phenotype-genotype correlation. Transl Sci Rare Dis. (2017) 2:157–64. 10.3233/TRD-170017

[284] Haghighi A Haack TB Atiq M Mottaghi H Haghighi-Kakhki H Bashir RA. Sengers syndrome: six novel AGK mutations in seven new families and review of the phenotypic and mutational spectrum of 29 patients. Orphanet J Rare Dis. (2014) 9:119. 10.1186/s13023-014-0119-3 25208612 PMC4167147

[285] Kang Y Stroud DA Baker MJ De Souza DP Frazier AE Liem M. Sengers syndrome-associated mitochondrial acylglycerol kinase is a subunit of the human TIM22 protein import complex. Mol Cell. (2017) 67:457–70 e5. 10.1016/j.molcel.2017.06.014 28712726

[286] Kor D Yilmaz B Horoz O Ceylaner G Sizmaz S Demir F. Two novel mutations in the AGK gene: two case reports with Sengers syndrome. Gene Technol. (2016) 5:2. 10.4172/2329-6682.1000140

[287] Dabir DV Hasson SA Setoguchi K Johnson ME Wongkongkathep P Douglas CJ. A small molecule inhibitor of redox-regulated protein translocation into mitochondria. Dev Cell. (2013) 25:81–92. 10.1016/j.devcel.2013.03.006 23597483 PMC3726224

[288] Berger I Ben-Neriah Z Dor-Wolman T Shaag A Saada A Zenvirt S. Early prenatal ventriculomegaly due to an AIFM1 mutation identified by linkage analysis and whole exome sequencing. Mol Genet Metab. (2011) 104:517–20. 10.1016/j.ymgme.2011.09.020 22019070

[289] Heimer G Eyal E Zhu X Ruzzo EK Marek-Yagel D Sagiv D. Mutations in AIFM1 cause an X-linked childhood cerebellar ataxia partially responsive to riboflavin. Eur J Paediatr Neurol. (2018) 22:93–101. 10.1016/j.ejpn.2017.09.004 28967629

[290] Joza N Oudit GY Brown D Benit P Kassiri Z Vahsen N. Muscle-specific loss of apoptosis-inducing factor leads to mitochondrial dysfunction, skeletal muscle atrophy, and dilated cardiomyopathy. Mol Cell Biol. (2005) 25:10261–72. 10.1128/MCB.25.23.10261-10272.2005 16287843 PMC1291246

[291] van Empel VP Bertrand AT van der Nagel R Kostin S Doevendans PA Crijns HJ. Downregulation of apoptosis-inducing factor in harlequin mutant mice sensitizes the myocardium to oxidative stress-related cell death and pressure overload-induced decompensation. Circ Res. (2005) 96:e92–e101. 10.1161/01.RES.0000172081.30327.28 15933268

[292] Xu A Szczepanek K Hu Y Lesnefsky EJ Chen Q. Cardioprotection by modulation of mitochondrial respiration during ischemia-reperfusion: role of apoptosis-inducing factor. Biochem Biophys Res Commun. (2013) 435:627–33. 10.1016/j.bbrc.2013.05.033 23685150

[293] Chen L Shi D Guo M. The roles of PKC-delta and PKC-epsilon in myocardial ischemia/reperfusion injury. Pharmacol Res. (2021) 170:105716. 10.1016/j.phrs.2021.105716 34102229

[294] Javadov S Karmazyn M Escobales N. Mitochondrial permeability transition pore opening as a promising therapeutic target in cardiac diseases. J Pharmacol Exp Ther. (2009) 330:670–8. 10.1124/jpet.109.153213 19509316

[295] Schleiff E Silvius JR Shore GC. Direct membrane insertion of voltage-dependent anion-selective channel protein catalyzed by mitochondrial Tom20. J Cell Biol. (1999) 145:973–8. 10.1083/jcb.145.5.973 10352015 PMC2133124

[296] Shi D Qi M Zhou L Li X Ni L Li C. Endothelial mitochondrial preprotein translocase Tomm7-Rac1 signaling axis dominates cerebrovascular network homeostasis. Arterioscler Thromb Vasc Biol. (2018) 38:2665–77. 10.1161/ATVBAHA.118.311538 30354240

[297] Richter-Dennerlein R Korwitz A Haag M Tatsuta T Dargazanli S Baker M. DNAJC19, a mitochondrial cochaperone associated with cardiomyopathy, forms a complex with prohibitins to regulate cardiolipin remodeling. Cell Metab. (2014) 20:158–71. 10.1016/j.cmet.2014.04.016 24856930

[298] Sinha D Joshi N Chittoor B Samji P D'Silva P. Role of Magmas in protein transport and human mitochondria biogenesis. Hum Mol Genet. (2010) 19:1248–62. 10.1093/hmg/ddq002 20053669 PMC2838536

[299] Vukotic M Nolte H Konig T Saita S Ananjew M Kruger M. Acylglycerol kinase mutated in sengers syndrome is a subunit of the TIM22 protein translocase in mitochondria. Mol Cell. (2017) 67:471–83 e7. 10.1016/j.molcel.2017.06.013 28712724

[300] Siriwardena K Mackay N Levandovskiy V Blaser S Raiman J Kantor PF. Mitochondrial citrate synthase crystals: novel finding in Sengers syndrome caused by acylglycerol kinase (AGK) mutations. Mol Genet Metab. (2013) 108:40–50. 10.1016/j.ymgme.2012.11.282 23266196

[301] Hangen E Feraud O Lachkar S Mou H Doti N Fimia GM. Interaction between AIF and CHCHD4 regulates respiratory chain biogenesis. Mol Cell. (2015) 58:1001–14. 10.1016/j.molcel.2015.04.020 26004228

[302] Meyer K Buettner S Ghezzi D Zeviani M Bano D Nicotera P. Loss of apoptosis-inducing factor critically affects MIA40 function. Cell Death Dis. (2015) 6:e1814. 10.1038/cddis.2015.170 26158520 PMC4650723

[303] Modjtahedi N Kroemer G. CHCHD4 links AIF to the biogenesis of respiratory chain complex I. Mol Cell Oncol. (2016) 3:e1074332. 10.1080/23723556.2015.1074332 27308594 PMC4905392

[304] Bertero E Kutschka I Maack C Dudek J. Cardiolipin remodeling in Barth syndrome and other hereditary cardiomyopathies. Biochim Biophys Acta Mol Basis Dis. (2020) 1866:165803. 10.1016/j.bbadis.2020.165803 32348916

[305] Monteiro JP Oliveira PJ Jurado AS. Mitochondrial membrane lipid remodeling in pathophysiology: a new target for diet and therapeutic interventions. Prog Lipid Res. (2013) 52:513–28. 10.1016/j.plipres.2013.06.002 23827885

[306] Paradies G Paradies V Ruggiero FM Petrosillo G. Role of cardiolipin in mitochondrial function and dynamics in health and disease: molecular and pharmacological aspects. Cells. (2019) 8:70728. 10.3390/cells8070728 31315173 PMC6678812

[307] Brandner K Mick DU Frazier AE Taylor RD Meisinger C Rehling P. Taz1, an outer mitochondrial membrane protein, affects stability and assembly of inner membrane protein complexes: implications for Barth Syndrome. Mol Biol Cell. (2005) 16:5202–14. 10.1091/mbc.e05-03-0256 16135531 PMC1266419

[308] Sabbah HN. Barth syndrome cardiomyopathy: targeting the mitochondria with elamipretide. Heart Fail Rev. (2021) 26:237–53. 10.1007/s10741-020-10031-3 33001359 PMC7895793

[309] Vreken P Valianpour F Nijtmans LG Grivell LA Plecko B Wanders RJ. Defective remodeling of cardiolipin and phosphatidylglycerol in Barth syndrome. Biochem Biophys Res Commun. (2000) 279:378–82. 10.1006/bbrc.2000.3952 11118295

[310] Gebert N Joshi AS Kutik S Becker T McKenzie M Guan XL. Mitochondrial cardiolipin involved in outer-membrane protein biogenesis: implications for Barth syndrome. Curr Biol. (2009) 19:2133–9. 10.1016/j.cub.2009.10.074 19962311 PMC4329980

[311] Kutik S Rissler M Guan XL Guiard B Shui G Gebert N. The translocator maintenance protein Tam41 is required for mitochondrial cardiolipin biosynthesis. J Cell Biol. (2008) 183:1213–21. 10.1083/jcb.200806048 19114592 PMC2606970

[312] Tamura Y Harada Y Yamano K Watanabe K Ishikawa D Ohshima C. Identification of Tam41 maintaining integrity of the TIM23 protein translocator complex in mitochondria. J Cell Biol. (2006) 174:631–7. 10.1083/jcb.200603087 16943180 PMC2064306

[313] Boengler K Schulz R. Connexin 43 and mitochondria in cardiovascular health and disease. Adv Exp Med Biol. (2017) 982:227–46. 10.1007/978-3-319-55330-6_12 28551790

[314] Michela P Velia V Aldo P Ada P. Role of connexin 43 in cardiovascular diseases. Eur J Pharmacol. (2015) 768:71–6. 10.1016/j.ejphar.2015.10.030 26499977

[315] Wang M Smith K Yu Q Miller C Singh K Sen CK. Mitochondrial connexin 43 in sex-dependent myocardial responses and estrogen-mediated cardiac protection following acute ischemia/reperfusion injury. Basic Res Cardiol. (2019) 115:1. 10.1007/s00395-019-0759-5 31741053 PMC7410524

[316] Boengler K Konietzka I Buechert A Heinen Y Garcia-Dorado D Heusch G. Loss of ischemic preconditioning's cardioprotection in aged mouse hearts is associated with reduced gap junctional and mitochondrial levels of connexin 43. Am J Physiol Heart Circ Physiol. (2007) 292:H1764–9. 10.1152/ajpheart.01071.2006 17142336

[317] Schulz R Boengler K Totzeck A Luo Y Garcia-Dorado D Heusch G. Connexin 43 in ischemic pre- and postconditioning. Heart Fail Rev. (2007) 12:261–6. 10.1007/s10741-007-9032-3 17516165

[318] Ruiz-Meana M Rodriguez-Sinovas A Cabestrero A Boengler K Heusch G Garcia-Dorado D. Mitochondrial connexin43 as a new player in the pathophysiology of myocardial ischaemia-reperfusion injury. Cardiovasc Res. (2008) 77:325–33. 10.1093/cvr/cvm062 18006437

[319] Saito T Sadoshima J. Molecular mechanisms of mitochondrial autophagy/mitophagy in the heart. Circ Res. (2015) 116:1477–90. 10.1161/CIRCRESAHA.116.303790 25858070 PMC4419704

[320] Mukherjee UA Ong SB Ong SG Hausenloy DJ. Parkinson's disease proteins: novel mitochondrial targets for cardioprotection. Pharmacol Ther. (2015) 156:34–43. 10.1016/j.pharmthera.2015.10.005 26481155 PMC4667215

[321] Friederich MW Erdogan AJ Coughlin CR Elos MT Jiang H O'Rourke CP. Mutations in the accessory subunit NDUFB10 result in isolated complex I deficiency and illustrate the critical role of intermembrane space import for complex I holoenzyme assembly. Hum Mol Genet. (2017) 26:702–16. 10.1093/hmg/ddw431 28040730 PMC6251674

[322] Chis R Sharma P Bousette N Miyake T Wilson A Backx PH. alpha-Crystallin B prevents apoptosis after H2O2 exposure in mouse neonatal cardiomyocytes. Am J Physiol Heart Circ Physiol. (2012) 303:H967–78. 10.1152/ajpheart.00040.2012 22904156 PMC3706333

[323] Zhang Y Li XR Zhao L Duan GL Xiao L Chen HP. DJ-1 preserving mitochondrial complex I activity plays a critical role in resveratrol-mediated cardioprotection against hypoxia/reoxygenation-induced oxidative stress. Biomed Pharmacother. (2018) 98:545–52. 10.1016/j.biopha.2017.12.094 29287203

[324] Deng YZ Xiao L Zhao L Qiu LJ Ma ZX Xu XW. Molecular mechanism underlying hypoxic preconditioning-promoted mitochondrial translocation of DJ-1 in hypoxia/reoxygenation H9c2 cells. Molecules. (2019) 25:10071. 10.3390/molecules25010071 31878239 PMC6983240

[325] Ding H Xu XW Wang H Xiao L Zhao L Duan GL. DJ-1 plays an obligatory role in the cardioprotection of delayed hypoxic preconditioning against hypoxia/reoxygenation-induced oxidative stress through maintaining mitochondrial complex I activity. Cell Biochem Funct. (2018) 36:147–54. 10.1002/cbf.3326 29431188

[326] Baseler WA Dabkowski ER Jagannathan R Thapa D Nichols CE Shepherd DL. Reversal of mitochondrial proteomic loss in Type 1 diabetic heart with overexpression of phospholipid hydroperoxide glutathione peroxidase. Am J Physiol Regul Integr Comp Physiol. (2013) 304:R553–65. 10.1152/ajpregu.00249.2012 23408027 PMC3627941

[327] Dabkowski ER Baseler WA Williamson CL Powell M Razunguzwa TT Frisbee JC. Mitochondrial dysfunction in the type 2 diabetic heart is associated with alterations in spatially distinct mitochondrial proteomes. Am J Physiol Heart Circ Physiol. (2010) 299:H529–40. 10.1152/ajpheart.00267.2010 20543078 PMC2930393

[328] Craig EE Hood DA. Influence of aging on protein import into cardiac mitochondria. Am J Physiol. (1997) 272:H2983–8. 10.1152/ajpheart.1997.272.6.H2983 9227577

[329] Schneider JJ Hood DA. Effect of thyroid hormone on mtHsp70 expression, mitochondrial import and processing in cardiac muscle. J Endocrinol. (2000) 165:9–17. 10.1677/joe.0.1650009 10750031

[330] Marin-Garcia J. Thyroid hormone and myocardial mitochondrial biogenesis. Vascul Pharmacol. (2010) 52:120–30. 10.1016/j.vph.2009.10.008 19857604

[331] Craig EE Chesley A Hood DA. Thyroid hormone modifies mitochondrial phenotype by increasing protein import without altering degradation. Am J Physiol. (1998) 275:C1508–15. 10.1152/ajpcell.1998.275.6.C1508 9843712

[332] Colavecchia M Christie LN Kanwar YS Hood DA. Functional consequences of thyroid hormone-induced changes in the mitochondrial protein import pathway. Am J Physiol Endocrinol Metab. (2003) 284:E29–35. 10.1152/ajpendo.00294.2002 12388124

